# Synthesis of thioethers, arenes and arylated benzoxazoles by transformation of the C(aryl)–C bond of aryl alcohols†

**DOI:** 10.1039/d0sc01229g

**Published:** 2020-04-15

**Authors:** Mingyang Liu, Zhanrong Zhang, Bingfeng Chen, Qinglei Meng, Pei Zhang, Jinliang Song, Buxing Han

**Affiliations:** Beijing National Laboratory for Molecular Science, CAS Key Laboratory of Colloid and Interface and Thermodynamics, CAS Research/Education Center for Excellence in Molecular Sciences, Institute of Chemistry, Chinese Academy of Sciences Beijing 100190 China zhangzhanrong@iccas.ac.cn hanbx@iccas.ac.cn; School of Chemistry and Chemical Engineering, University of Chinese Academy of Sciences Beijing 100049 China; Physical Science Laboratory, Huairou National Comprehensive Science Center Beijing 101400 China

## Abstract

Transformation of aryl alcohols into high-value functionalized aromatic compounds by selective cleavage and functionalization of the C(aryl)–C(OH) bond is of crucial importance, but very challenging by far. Herein, for the first time, we report a novel and versatile strategy for activation and functionalization of C(aryl)–C(OH) bonds by the cooperation of oxygenation and decarboxylative functionalization. A diverse range of aryl alcohol substrates were employed as arylation reagents *via* the cleavage of C(aryl)–C(OH) bonds and effectively converted into corresponding thioether, arene, and arylated benzoxazole products in excellent yields, in a Cu based catalytic system using O_2_ as the oxidant. This study offers a new way for aryl alcohol conversion and potentially offers a new opportunity to produce high-value functionalized aromatics from renewable feedstocks such as lignin which features abundant C(aryl)–C(OH) bonds in its linkages.

## Abstract 2

Aryl alcohols served as aryl agents by effective activation of C(aryl)–C(OH) bonds, and were transformed into corresponding thioether, arene, and arylated benzoxazole products in excellent yields.

## Graphical abstract

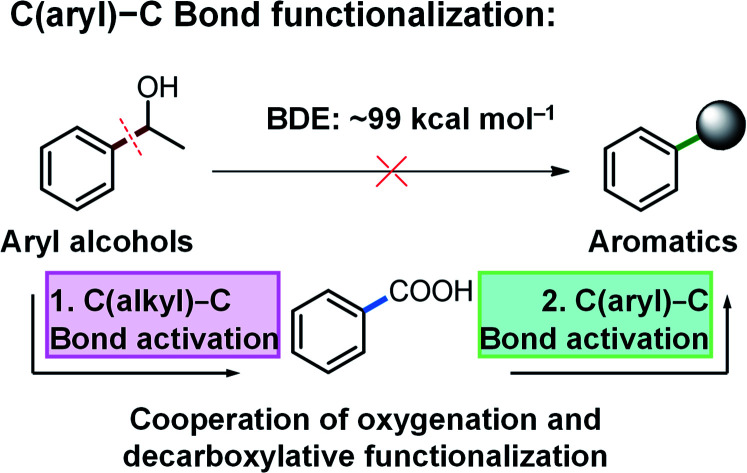

## Introduction

**Fig. 1:**
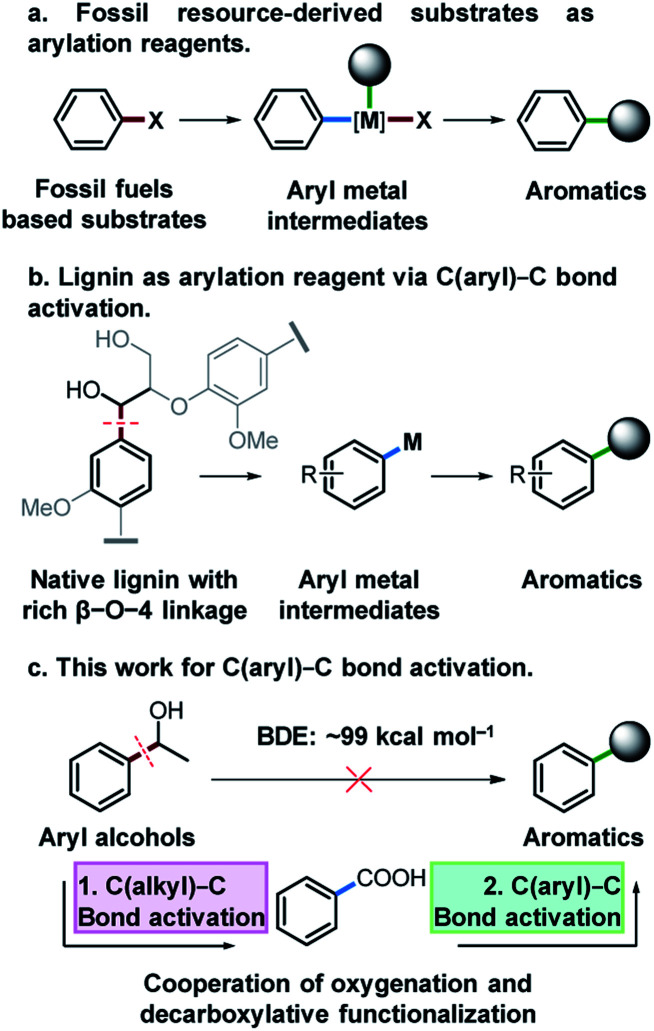
Research background and design. (a) Traditional arylation reaction by using fossil fuel-based substrates. (b) Envisioned strategy for using lignin as the arylating reagent. (c) Our proposed Cu catalyzed arylation by using aryl alcohols as arylating agents mediated with acid intermediates.

Functionalized aromatic compounds are ubiquitously involved in and of significant importance in medicinal chemistry, biological chemistry and the chemical industry. Selective production of aromatic compounds represents one of the most attractive and imperative topics in organic and synthetic chemistry.^1^ In this regard, research advances on cross-coupling reactions have significantly promoted this area and these methods are now routinely employed in both fundamental research and industry.^2–5^ Notably, significant efforts have been devoted to constructing aryl/alkyl-metal intermediates (Fig. 1a) for cross-coupling reactions to produce aromatic compounds with diverse functionalities. However, there are still many challenges that need to be addressed in this field. For instance, expensive pre-functionalized aromatic substrates originating from finite and depleting fossil resources, such as aryl halides,^6^ aniline/thiophenol/phenol derivatives^7–15^ and aryl compounds,^16–25^ are almost exclusively used as feedstocks. Meanwhile, cross-coupling reactions suffer from some drawbacks such as low atom economy, requirement for sophisticated catalysts or catalytic systems, generation and inevitable handling of toxic and corrosive halide-containing wastes, *etc.* To address these issues, on one hand, it is crucial to develop more efficient catalytic systems with improved atom economy for cross-coupling reactions. On the other hand, it is important to divert the current fossil-based feedstocks to naturally renewable biomass resources.

Lignin represents the only naturally renewable aromatic resource and features abundant C(aryl)–C(OH) structures in its linkage motifs (occurrence values of β-O-4 type linkages: 45–84%),^26–29^ and the activation of the C(aryl)–C(OH) bond to construct aryl-metal intermediates of phenyl ethanol-based compounds offers an attractive strategy for both lignin depolymerization and functionalization to produce fine functionalized aromatic chemicals (Fig. 1b). Among the various types of ubiquitous C–C bonds, C(aryl)–C bonds are relatively less polar and possess higher stability (bond dissociation energy: ∼99 kcal mol^−1^).^30^ Their direct activation and functionalization are kinetically inert. In this regard, very limited research advances have been achieved by far and wide application of developed methods is often hindered, owing to the requirement of indispensable directing group modified substrates,^31–34^ highly toxic reagents and strong oxidants.^30,35^ The development of a versatile and robust strategy for the activation and functionalization of C(aryl)–C bonds that allows aryl alcohols to be used as latent arylating agents is highly desirable, but very challenging. Notably, in the pursuit of a sustainable future and biobased economy, this also represents a very interesting topic in terms of effectively transforming the only naturally renewable aromatic resource lignin into high-value fine aromatic chemicals.

Catalytic oxidation is an effective method for the cleavage of C(alkyl)–C bonds to generate acid^36,37^ or aldehyde products.^38^ Meanwhile, aryl acids could be used as arylating agents for decarboxylative coupling reactions which is an effective synthetic strategy for the activation of the C(aryl)–C bond with less toxic by-products.^16–18,23^ Herein, for the first time, we report activation and functionalization of C(aryl)–C(OH) bonds of aryl alcohols to produce thioethers, arenes and arylated benzoxazole products by the cooperation of oxygenation and decarboxylative functionalization (Fig. 1c). Using aryl alcohols as substrates and arylation reagents *via* the cleavage of C(aryl)–C(OH) bonds, and environmentally benign O_2_ as the oxidant, a diverse array of substrates were converted into the desired products with excellent yields in a Cu based catalytic system.

## Results and discussion

We started our study about C(aryl)–C(OH) bond activation and functionalization by oxidative thioetherification using a secondary aryl alcohol 1-(2-nitrophenyl)ethanol (**1a**) as a model substrate, in a catalytic reaction system consisting of diphenyldisulfane (**2a**) as the sulfur source, CuSO_4_ as the catalyst, 1,10-phenanthroline as the ligand, K_2_CO_3_ as a base additive, DMSO as the solvent and O_2_ as the environmentally benign oxidant (Table 1). Initially, the targeted thioether product **3a** was generated in only 33% yield at 140 °C for 12 h reaction (entry 1). We then conducted a series of experiments to optimize the reaction conditions and improve the yield. For optimization details please refer to Fig. S1–S5.† Notably, it was found that using other polar aprotic solvent DMF (entry 2), nonpolar solvent toluene (entry 3), base additives (entries 4–5) and Cu salts (entries 6–8) resulted in lower yields of the thioether product (0–27%), while increasing the catalyst amount slightly improved the yield to 43% (entry 9). To our delight, when the amount of ligand was reduced, the yield of **3a** significantly improved to 93% (entry 10), indicating that a high ratio of metal to ligand (Cu : phen = 2 : 1) was crucial to this chemical transformation.

**Table 1:** Optimization of reaction conditions^a^

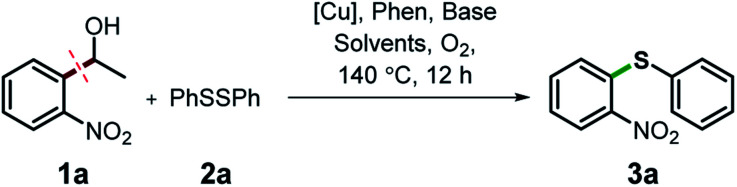					
Entry	[Cu]	Phen	Base	Solvent	Yield
1	30% CuSO_4_	40%	K_2_CO_3_	DMSO	33%
2	30% CuSO_4_	40%	K_2_CO_3_	DMF	6%
3	30% CuSO_4_	40%	K_2_CO_3_	Tol	0%
4	30% CuSO_4_	40%	Cs_2_CO_3_	DMSO	11%
5	30% CuSO_4_	40%	NaHCO_3_	DMSO	17%
6	30% Cu(Ac)_2_	40%	K_2_CO_3_	DMSO	27%
7	30% CuCl	40%	K_2_CO_3_	DMSO	4%
8	30% CuCl_2_	40%	K_2_CO_3_	DMSO	9%
9	40% CuSO_4_	40%	K_2_CO_3_	DMSO	43%
10	40% CuSO_4_	20%	K_2_CO_3_	DMSO	93%

a Experiments were performed on the 0.2 mmol scale unless otherwise noted. Reaction conditions: 0.2 mmol 1-(2-nitrophenyl)ethanol, 0.4 mmol diphenyldisulfane, 0.06 or 0.08 mmol [Cu] salt, 0.08 or 0.04 mmol anhydrous 1,10-phenanthroline (phen), 1 mmol base, 200 mg 4 Å molecular sieve (4 Å), 2 mL solvent, 0.5 MPa O_2_, 140 °C, 12 h. Yield was determined by gas chromatography (GC).

Next, the scope of this oxidative C(aryl)–C(OH) bond thioetherification reaction was examined in detail under the optimized reaction conditions (Table 2). Benzyl alcohol **1b** and alkyl alcohol **1c** were effectively converted into thioether products in excellent yields (95–97%). Secondary alcohols with various alkyl carbon-chain lengths (**1a**, **1d**) also reacted smoothly. 1-(2-Nitrophenyl)ethanol derivatives with electron-donating or electron-withdrawing substituents on both the aryl ring (**1h–1k**) and the methyl group (**1l–1n**) are also suitable substrates, affording corresponding thioether products in moderate to excellent yields (63–95%). In addition, this transformation could also tolerate heteroaryl alcohol substrates (**1o–1q**) and asymmetric thioether products were obtained in satisfactory yields. Moreover, cellulosic biomass-derived furan compounds (**1r–1t**) performed well, affording corresponding thioether products in high yields (69–89%). In addition to using diphenyldisulfane as the sulfur source, we also explored other sulfur sources including diphenyldiselane (**2b**) and diphenyldisulfane derivatives with both electron-donating (**2d**) and electron-withdrawing substituent groups (**2c**) on the aryl ring, under optimized reaction conditions (Fig. S6†). These sulfur sources were also tolerated and effectively transformed into corresponding products. Other sulfur sources including dibenzyldisulfane (**2e**) or aliphatic dihexyldisulfane (**2f**) are not satisfactory in this reaction, probably because of the weaker coordination to the Cu catalyst in comparison to that between the Cu catalyst and aryl disulphide we used (Fig. S6†).

**Table 2:** Substrate scope for the oxidative transformation of aryl alcohols to thioethers^a^

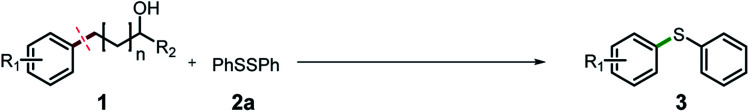					
Substrates	Products	Yield	Substrates	Products	Yield
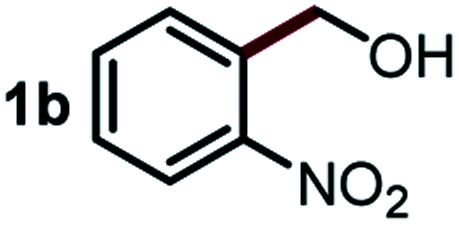	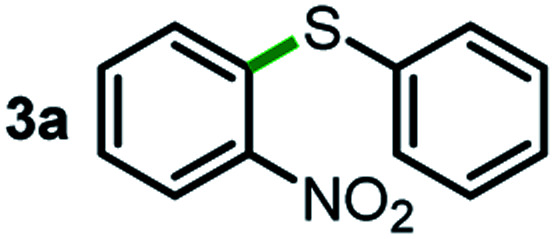	97%	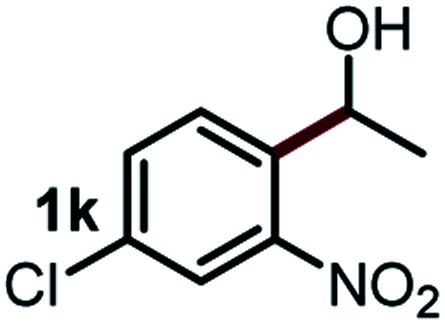	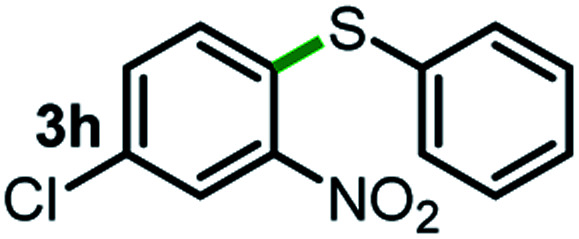	79%*
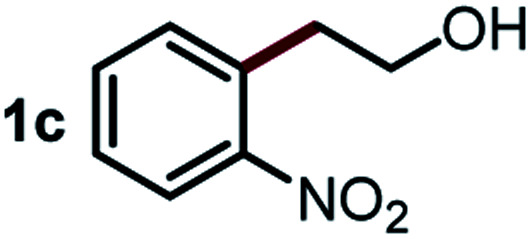	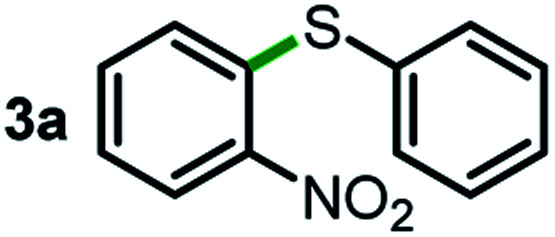	95%	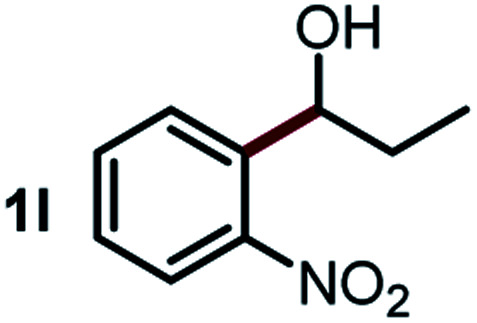	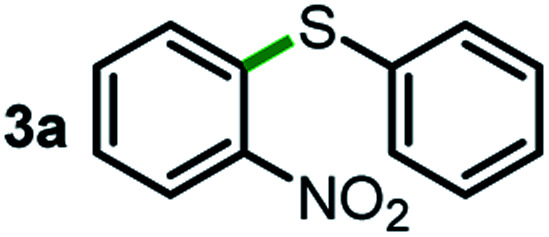	95%
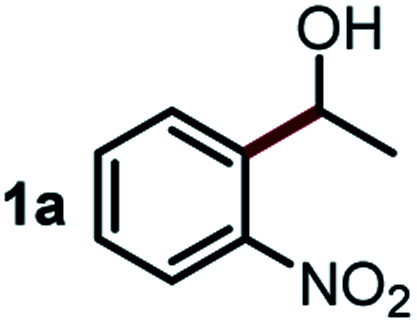	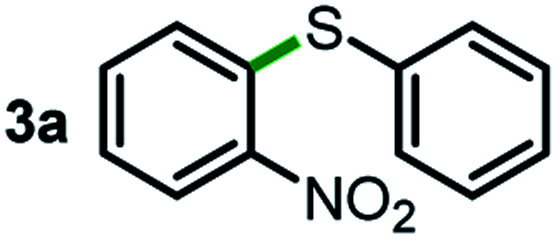	93%	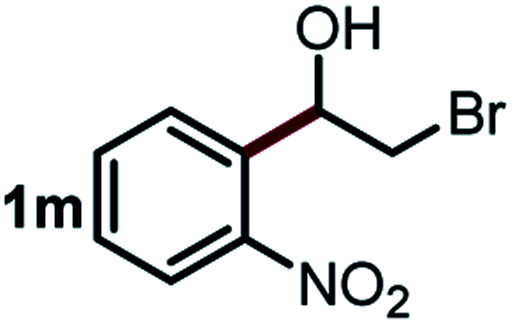	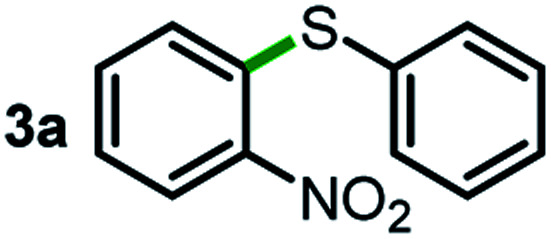	63%
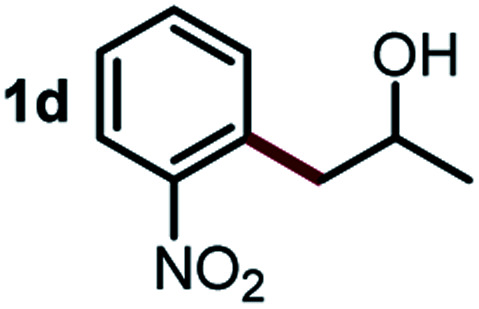	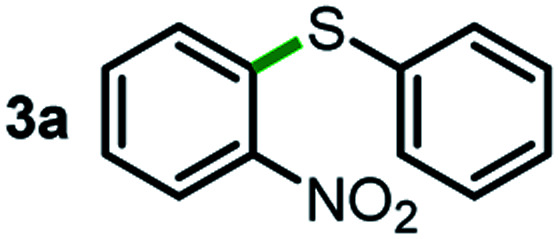	87%	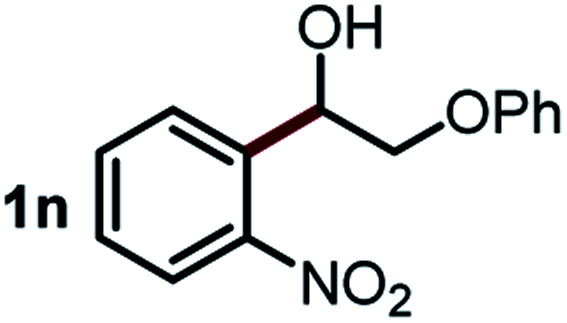	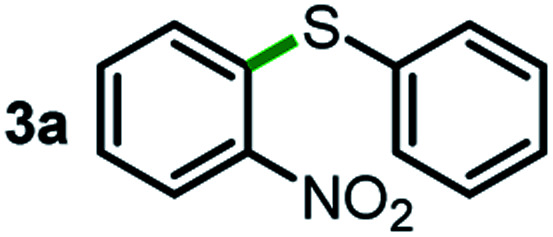	88%
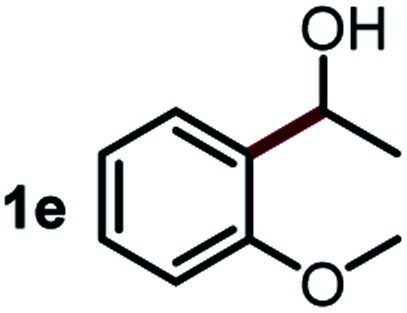	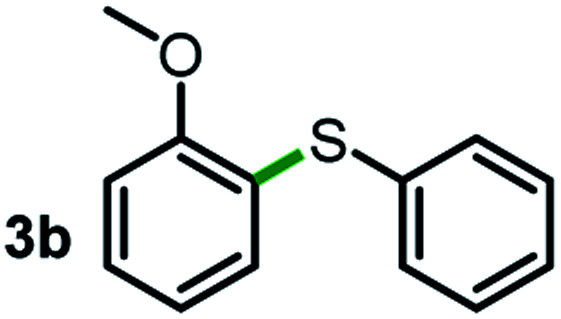	22%	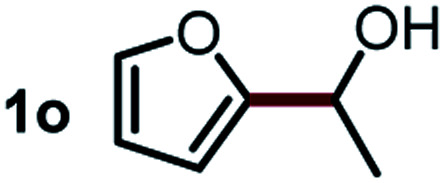	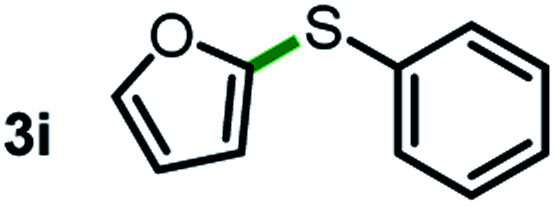	72%*
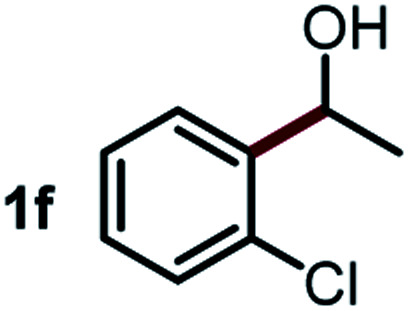	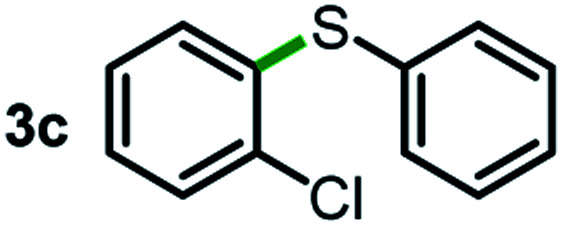	32%	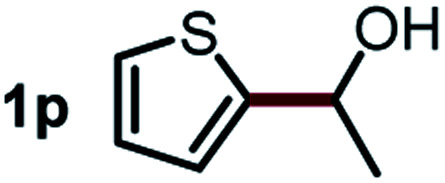	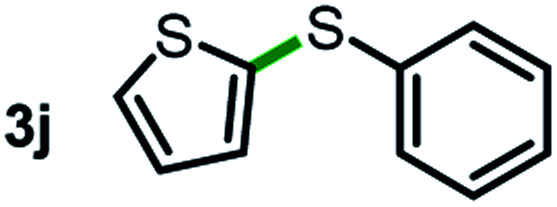	86%*
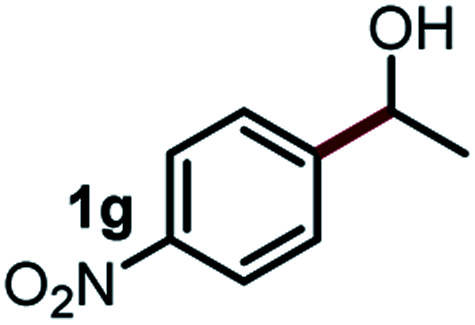	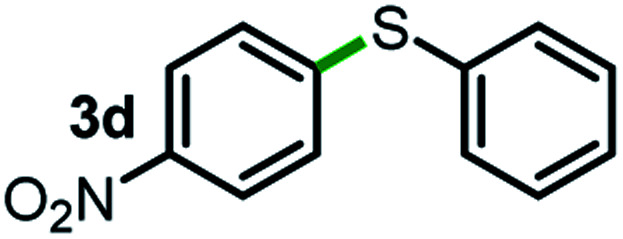	5%*	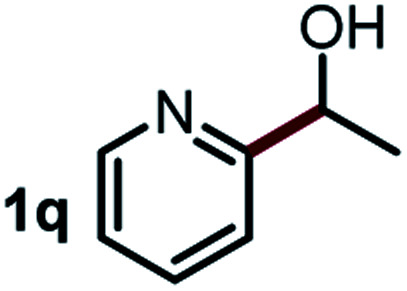	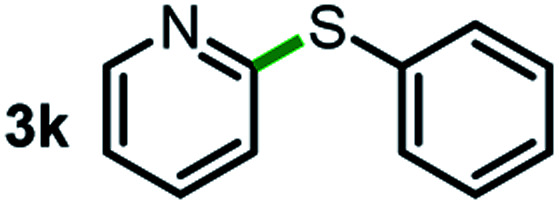	80%*
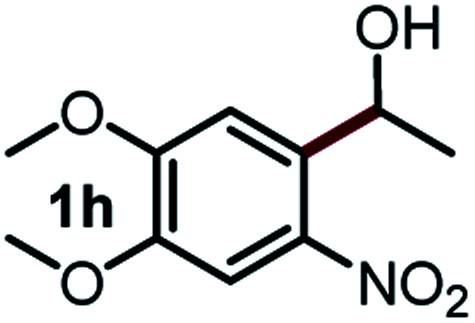	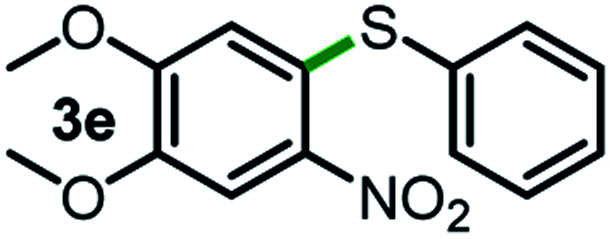	90%*	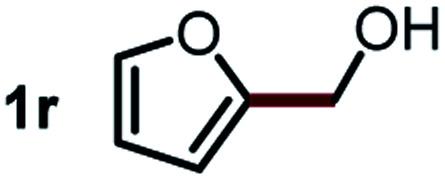	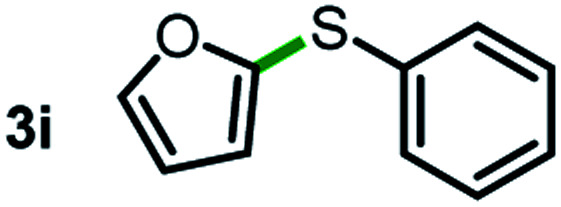	89%*
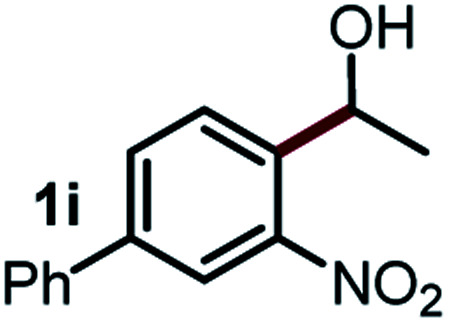	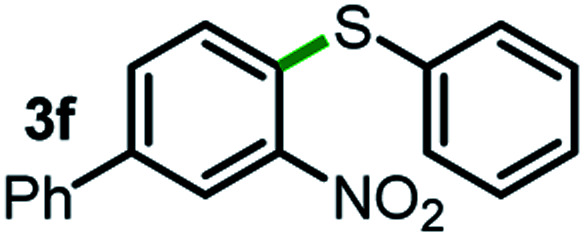	93%*	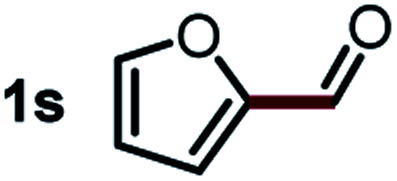	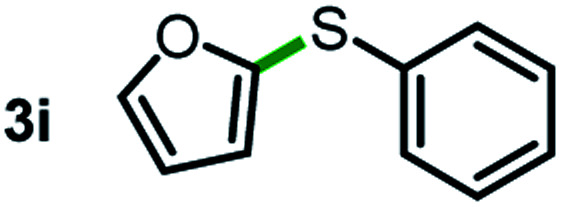	87%*
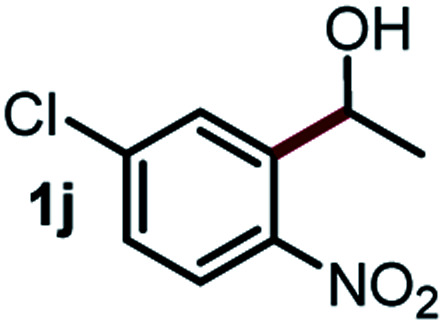	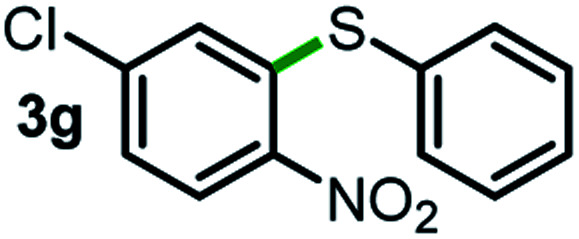	79%*	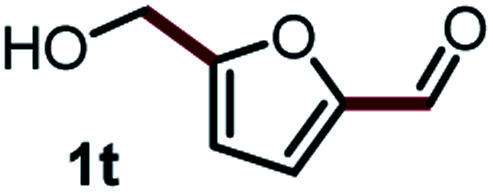	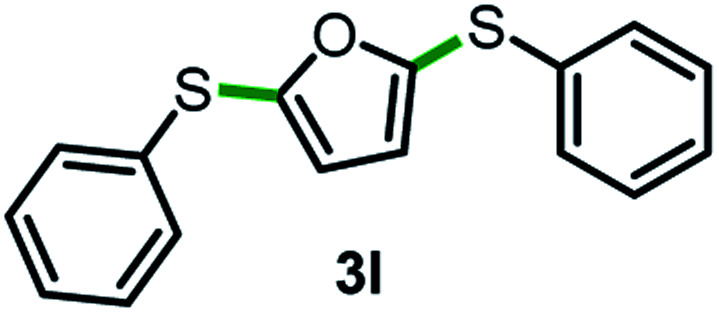	69%*

a Experiments were performed on the 0.2 mmol scale unless otherwise noted. Optimized conditions: 0.2 mmol alcohols, 0.4 mmol diphenyldisulfane, 0.08 mmol CuSO_4_, 0.04 mmol phen, 0.8 mmol K_2_CO_3_, 200 mg 4 Å, 2 mL DMSO, 0.5 MPa O_2_, 140 °C, 12 h. *24 h. Yield was determined by GC.

**Fig. 2:**
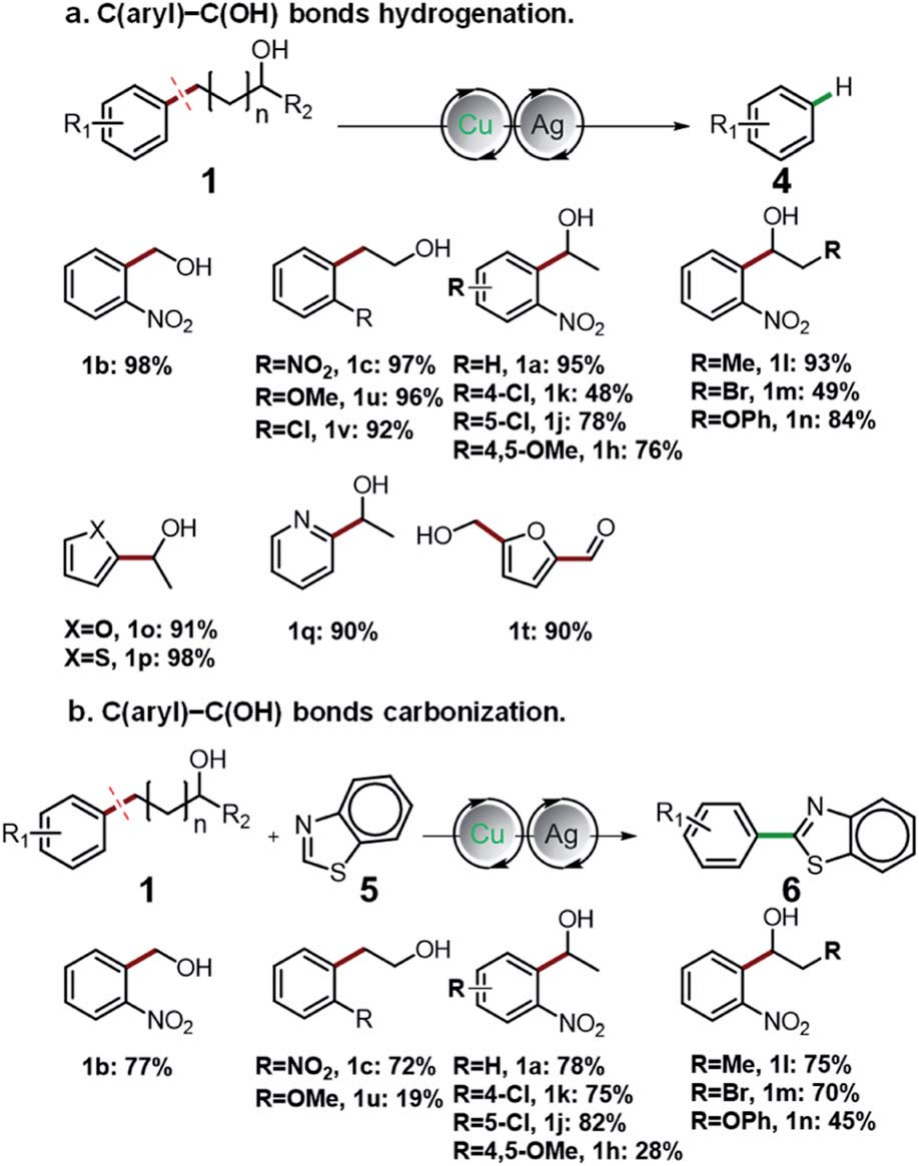
Universality of C(aryl)–C(OH) bond functionalization. (a) C(aryl)–C(OH) bond hydrogenation of aryl alcohols. (b) C(aryl)–C(OH) bonds carbonization of aryl alcohols.

Having thoroughly investigated the oxidative cleavage and thioetherification reaction, we further investigated the C(aryl)–C(OH) bond hydrogenation and carbonization of aryl alcohols (Fig. 2). By adding Ag salts into the standard catalytic system, the C(aryl)–C bonds of a diverse range of primary and secondary benzyl alcohols, as well as alkyl alcohols were selectively cleaved and functionalized to C(aryl)–H bonds, producing arenes in moderate to excellent yields ranging from 48% to 97% (Fig. 2a). Furthermore, for the first time, we employed aryl alcohols as aryl agents for regiospecific construction of aryl–heteroaryl bonds *via* C(aryl)–C(OH) bond carbonization (Fig. 2b). Under given conditions, the C(aryl)–C bonds and C–H bonds were simultaneously activated, affording aryl-benzothiazole products that possess important applications.^39,40^ For detailed information about the optimization of C(aryl)–C(OH) bond hydrogenation and carbonization reaction conditions, please refer to Fig. S7–S16.†

**Fig. 3:**
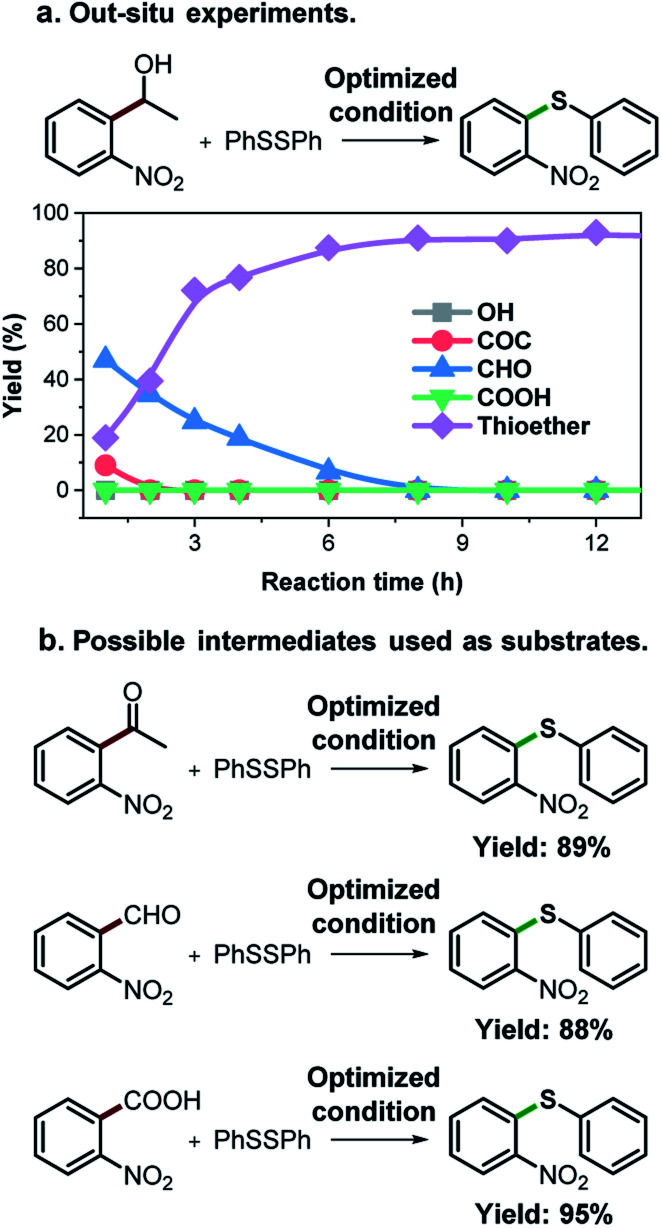
Mechanism study. (a) *ex situ* experiments recorded the evolution of intermediates of the oxidative C(aryl)–C(OH) bond thioetherification reaction. OH: 1-(2-nitrophenyl)ethanol, COC: 1-(2-nitrophenyl)ethenone, CHO: 2-nitrobenzaldehyde, COOH: 2-nitrobenzoic acid. (b) Potential intermediates were tested under the optimized conditions (the same as Table 2).

As an effort to explore the reaction mechanism, we then probed the potential intermediates during the oxidative thioetherification reaction (Fig. 3). An *ex situ* kinetic study revealed that the ketone intermediate was generated at the very beginning of the reaction (1 h) and consumed quickly. Aldehyde was identified as the major intermediate and present for several hours of reaction (Fig. 3a). To get further in-depth details, possible intermediates including ketone, aldehyde and acid were tested under optimized conditions. In this case, these possible intermediates were all effectively converted into the thioether product in excellent yields (Fig. 3b). These control experiments indicate that the oxidative C(aryl)–C(OH) bond thioetherification reaction was mediated by dehydrogenation of alcohol to ketone, C(C=O)–C bond cleavage of ketone to aldehyde, and oxidation of aldehyde to acid, together with decarboxylative thioetherification of acid intermediates. Since acid intermediates were not detected in the *ex situ* experiments, the oxidation of aldehyde to acid intermediates, rather than the decarboxylation step, represents the rate-determining step. This effect is also verified by ESI experiments (Fig. S17†).

**Fig. 4:**
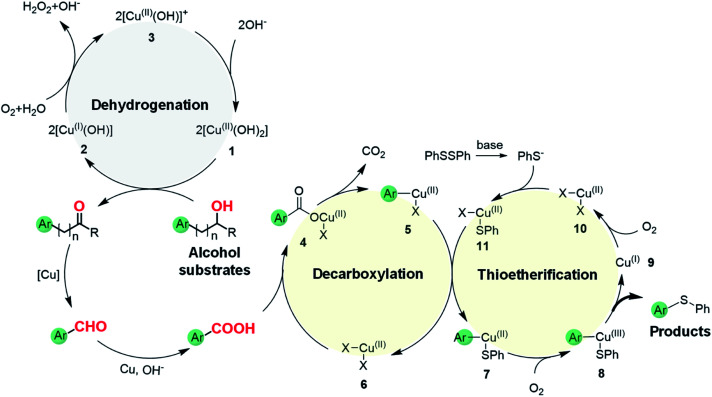
Plausible reaction pathway for the oxidative C(aryl)–C(OH) bond thioetherification reaction.

Based on the above results, we propose a plausible reaction mechanism for the oxidative C(aryl)–C(OH) bond thioetherification reaction using aryl alcohols as arylating agents (Fig. 4). The reaction is initiated by Cu/phenanthroline catalyzed dehydrogenation of the alcohol substrate under alkaline conditions. Deprotonation followed by hydride transfer of the alcohol substrate generates a ketone intermediate (R = H, aldehyde intermediate). Simultaneously, Cu^II^ species **1** is reduced to Cu^I^**2** which then reacts with dioxygen to generate the Cu^II^**3** intermediate. Under alkaline conditions, Cu^II^ species **1** is regenerated from the Cu^II^**3** with consumption of OH^−^.^41^ Then, according to our previous research,^42,43^ Cu catalyzed successive C(CO)–C bond cleavage of the ketone intermediate leads to an aryl aldehyde intermediate *via* hydroxylation of β-H and 1,2-hydride shift. The β-carbon of the alkyl substituent would transfer into formic acid and decompose to CO_2_ and H_2._ For the substrates containing phenoxy groups on the β-carbon (**1n**), the leaving part would be converted into phenyl formate followed by decomposition into benzoquinone, CO_2_ and H_2_ according to previous reports.^38,42–44^ In the presence of the Cu catalyst and under alkaline conditions, the aryl aldehyde is oxidized to aryl acid which represents the key intermediate for the thioetherification reaction.^45–47^ Subsequently, in the decarboxylation cycle, the acid–base reaction between aryl acid and Cu salts generates metal carboxylate **4**, which is further converted to aryl metal species **5***via* decarboxylation. Transmetalation between **5** and **11** generates a bifunctional aryl metal species **7** and active Cu^II^ species **6**. Thereafter, the final thioether product is generated by oxidation of **7** and reductive elimination of **8** in the thioetherificative cycle. It is noteworthy that aryl alcohol substrates serve as electrophilic synthetic equivalents for the C(aryl)–C(OH) bond thioetherification reaction. Notably, all three reactions are mediated with acid intermediates, and probable reaction pathways for hydrogenation and carbonization of C(aryl)–C(OH) bonds are illustrated in Fig. S18 and S19,† respectively.

## Conclusions

In summary, we have achieved functionalization including thioetherification, hydrogenation and carbonization of the C(aryl)–C(OH) bonds of aryl alcohols, using cheap copper salts as catalysts and oxygen as the environmentally benign oxidant. A diverse range of aryl alcohol substrates were used as arylation reagents and effectively converted into corresponding thioethers, arenes and arylated benzoxazole products in excellent yields. A detailed mechanism study indicated that this transformation was achieved by a well-ordered cooperation of oxygenation and decarboxylative functionalization both of which were catalyzed by the same Cu catalyst. In the process, oxidative cleavage of the C(alkyl)–C(OH) bond of alcohol substrates leads to aryl acid intermediates. Then decarboxylative functionalization is achieved *via* C(aryl)–C activation. Although at present this transformation is limited to *ortho*-substituted substrates (*e.g.*, *ortho*-nitroaryl alcohols), the current work provides a new route for oxidative cleavage and functionalization of the C(aryl)–C bond, and potentially offers a new way to produce functionalized aromatic compounds from renewable resources such as lignin. In this regard, further work is currently ongoing.

## Experimental

### Chemicals

Commercially available Cu salts (CuSO_4_, Cu(Ac)_2_, CuCl, CuCl_2_), Ag salts (Ag_2_O, Ag_2_CO_3_, AgCl, AgAc), bases (K_2_CO_3_, Cs_2_CO_3_, Na_2_CO_3_, NaOH, KOH, NaHCO_3_), 4 Å molecular sieves (4 Å), and anhydrous 1,10-phenanthroline (phen) were purchased from Acros, J&K, or Alfa Aesar. Diphenyldisulfanes, diphenyldiselane, benzoxazole, and solvents were purchased from Sigma-Aldrich. Alcohol substrates including 1-(2-nitrophenyl)ethanol (**1a**), (2-nitrophenyl)methanol (**1b**), 2-(2-nitrophenyl)ethanol (**1c**), 1-(2-nitrophenyl)propan-2-ol (**1d**), 1-(2-methoxyphenyl)ethanol (**1e**), 1-(2-chlorophenyl)ethanol (**1f**), 1-(3-nitro-[1,1′-biphenyl]-4-yl)ethanol (**1i**), 1-(2-nitrophenyl)propan-1-ol (**1l**), 1-(furan-2-yl)ethanol (**1o**), 1-(pyridin-2-yl)ethanol (**1q**), furan-2-ylmethanol (**1r**), furan-2-carbaldehyde (**1s**), and 5-(hydroxymethyl)furan-2-carbaldehyde (**1t**) were obtained from Sigma-Aldrich or Acros. Other alcohol substrates including **1p**, **1h**, **1k**, **1m**, **1n**, **1j**, and **1g** were synthesized according to the following procedure.

### Synthesis of alcohol substrates^48^

Aryl alcohols were synthesized by reduction of corresponding ketones with NaBH_4_ in MeOH. Typically, ketone (15 mmol) was dissolved in 100 mL MeOH and the solution was cooled to 0 °C in an ice bath for 10 min. NaBH_4_ (5 mmol) powder was added in several portions. The reaction was stirred at 0 °C for 10 min and warmed to room temperature. Thin layer chromatography (TLC) was used to record the transformation. Then the reaction was fully concentrated *in vacuo*, before adding 100 mL saturated aqueous NH_4_Cl. The organic products were extracted from the aqueous phase using ethyl acetate three times (3 × 100 mL). Finally, the organic phase was washed with brine, dried with anhydrous Na_2_SO_4_, and fully concentrated *in vacuo* to generate the corresponding alcohols. A further purification process was performed using gel column chromatography if necessary.

### Thioetherification reaction

0.2 mmol alcohol substrate, 0.4 mmol diphenyldisulfane, 0.08 mmol CuSO_4_, 0.04 mmol phen, 1 mmol K_2_CO_3_, 200 mg 4 Å, 0.2 mmol *n*-decane as the internal standard, and 2 mL DMSO were added into a Teflon-lined stainless-steel reactor, followed by charging 0.5 MPa O_2_ and heating at 140 °C for 12 h. After reaction, the reactor was quenched in an ice-water bath. Then, the reaction mixture was extracted using ethyl acetate and saturated aqueous NH_4_Cl to separate the products. Subsequently, the organic matter was further extracted using ethyl acetate twice and combined for qualitative and quantitative analysis.

### Hydrogenation reaction

0.2 mmol alcohol substrate, 0.12 mmol Cu(Ac)_2_, 0.08 mmol AgNO_3_, 0.08 mmol phen, 0.8 mmol NaOH, 0.2 mmol *n*-decane as the internal standard, and 2 mL DMSO were added into a Teflon-lined stainless-steel reactor, followed by charging 0.5 MPa O_2_ and heating at 140 °C for 12 h. After reaction, the reactor was quenched in an ice-water bath. Then, the reaction mixture was extracted using ethyl acetate and saturated aqueous NH_4_Cl to separate the products. Subsequently, the organic matter was further extracted using ethyl acetate twice and combined for qualitative and quantitative analysis.

### Carbonization reaction

0.2 mmol alcohol substrate, 0.5 mmol benzothiazole, 0.15 mmol CuCl, 0.25 mmol Ag_2_O, 0.1 mmol phen, 100 mg 4 Å, 0.2 mmol Cs_2_CO_3_, 0.2 mmol *n*-decane as the internal standard, and 2 mL DMSO were added into a Teflon-lined stainless-steel reactor, followed by charging 0.5 MPa O_2_ and heating at 140 °C for 12 h. After reaction, the reactor was quenched in an ice-water bath. Then, the reaction mixture was extracted using ethyl acetate and saturated aqueous NH_4_Cl to separate the products. Subsequently, the organic matter was further extracted with ethyl acetate twice and combined for qualitative and quantitative analysis.

### Characterization methods

GC-MS (Agilent 5975C-7890A, equipped with an electron ionization mass spectrometry detector, EI-MS) was used for qualitative analysis of products and other intermediates. The conversion of substrates and the yield of products were quantitatively analyzed using GC (Agilent 7820, equipped with a hydrogen flame-ionization detector, HP-5 non-polar columns) based on internal standard curves and areas of integrated peak area of corresponding species. NMR spectra was recorded on a Bruker Avance 400 spectrometer equipped with 5 mm pulsed-field-gradient (PFG) probes. DMSO-d_6_ or CDCl_3_ was used as the solvent. The resonance band of TMS or solvents was used as the internal standard. The spectra were recorded at 303 K.

## Conflicts of interest

There are no conflicts to declare.

## Supplementary Material

SC-011-D0SC01229G-s001
