# Supplementary material for: Synthesis of thioethers, arenes and arylated benzoxazoles by transformation of the C(aryl)–C bond of aryl alcohols
Source: Chem Sci. 2020 Apr 15;11(29):7634–40. doi: 10.1039/d0sc01229g (PMC8159426; doi:10.1039/d0sc01229g)
Supplement: SC-011-D0SC01229G-s001 [file SC-011-D0SC01229G-s001.pdf]

Electronic Supplementary Material (ESI) for Chemical Science.
This journal is © The Royal Society of Chemistry 2020

Supporting results

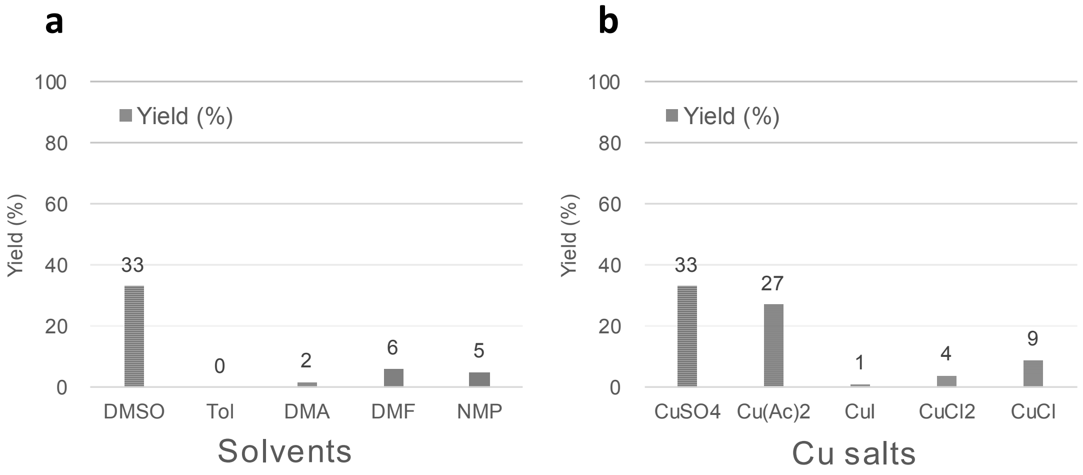

Figure S1. The effects of solvents and Cu salt catalysts on the C(aryl)−C bond thioetherification reaction. Reaction condition **a**: 0.2 mmol substrate **1a**, 0.4 mmol diphenyldisulfane, 0.06 mmol $CuSO_4$, 0.08 mmol phen, 0.8 mmol K_2CO_3, 200 mg 4Å, 2 mL **solvent**, 0.5 MPa O_2, 140 °C, 12 h. Reaction condition **b**: 0.2 mmol substrate **1a**, 0.4 mmol diphenyldisulfane, 0.06 mmol **Cu salts**, 0.08 mmol phen, 0.8 mmol K_2CO_3, 200 mg 4Å, 2 mL DMSO, 0.5 MPa O_2, 140 °C, 12 h.

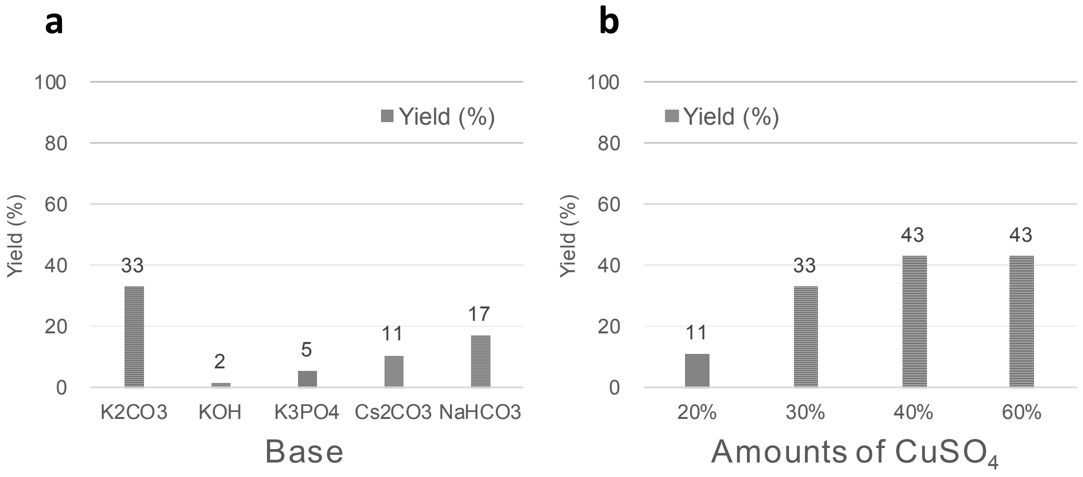

Figure S2. The effects of base and amount of $CuSO_4$ on the C(aryl)−C bond thioetherification reaction. Reaction condition **a**: 0.2 mmol substrate **1a**, 0.4 mmol diphenyldisulfane, 0.06 mmol $CuSO_4$, 0.08 mmol phen, **0.8 mmol base**, 200 mg 4Å, 2 mL DMSO, 0.5 MPa O_2, 140 °C, 12 h. Reaction condition **b**: 0.2 mmol substrate **1a**, 0.4 mmol diphenyldisulfane, **0.04-0.12 mmol $CuSO_4$**, 0.08 mmol phen, 0.8 mmol K_2CO_3, 200 mg 4Å, 2 mL DMSO, 0.5 MPa O_2, 140 °C, 12 h.

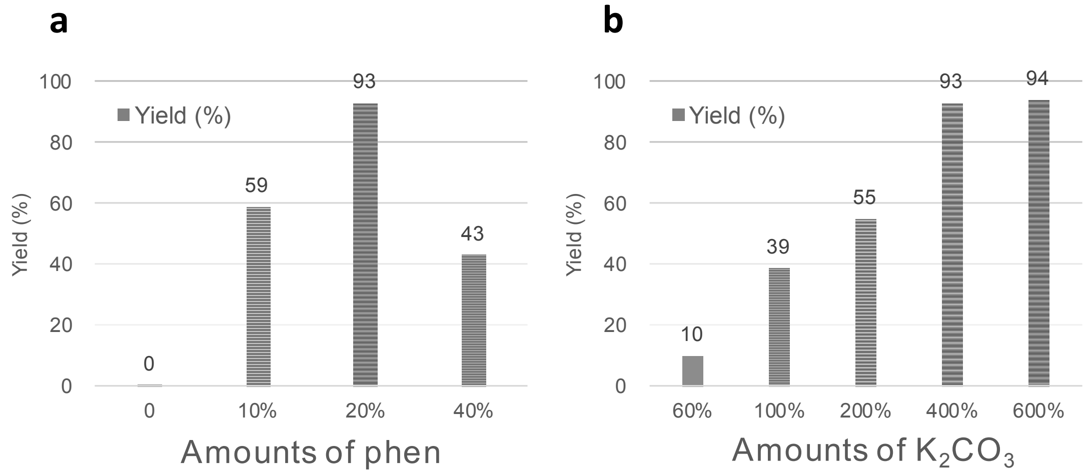

Figure S3. The effects of amounts of phen and K_2CO_3 on the C(aryl)−C bond thioetherification reaction. Reaction condition **a**: 0.2 mmol substrate **1a**, 0.4 mmol diphenyldisulfane, 0.08 mmol $CuSO_4$, **0-0.08 mmol phen**, 0.8 mmol K_2CO_3, 200 mg 4Å, 2 mL DMSO, 0.5 MPa O_2, 140 °C, 12 h. Reaction condition **b**: 0.2 mmol substrate **1a**, 0.4 mmol diphenyldisulfane, 0.08 mmol $CuSO_4$, 0.04 mmol phen, **0.12-1.2 mmol K_2CO_3**, 200 mg 4Å, 2 mL DMSO, 0.5 MPa O_2, 140 °C, 12 h.

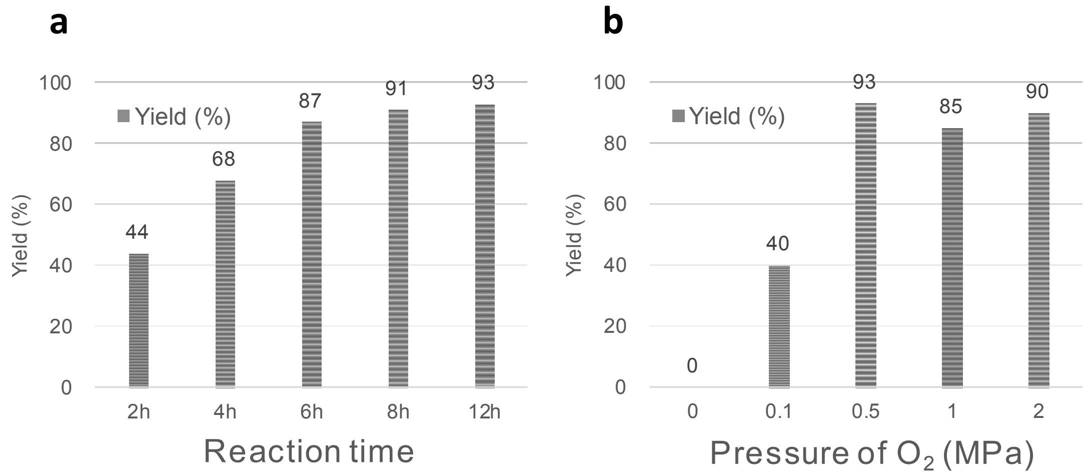

Figure S4. The Effect of reaction time and pressure of O_2 on the C(aryl)−C bond thioetherification reaction. Reaction condition **a**: 0.2 mmol substrate **1a**, 0.4 mmol diphenyldisulfane, 0.08 mmol $CuSO_4$, 0.04 mmol phen, 0.8 mmol K_2CO_3, 200 mg 4Å, 2 mL DMSO, 0.5 MPa O_2, 140 °C, **2-12 h**. Reaction condition **b**: 0.2 mmol substrate **1a**, 0.4 mmol diphenyldisulfane, 0.08 mmol $CuSO_4$, 0.04 mmol phen, 0.8 mmol K_2CO_3, 200 mg 4Å, 2 mL DMSO, **0-2 MPa O_2**, 140 °C, 12 h.

The reactions were carried out in a stainless-steel reactor with 10 mL Teflon tube. 5 atm O_2 is required to guarantee enough solute O_2 in DMSO, which is necessary to achieve satisfactory catalytic performances.

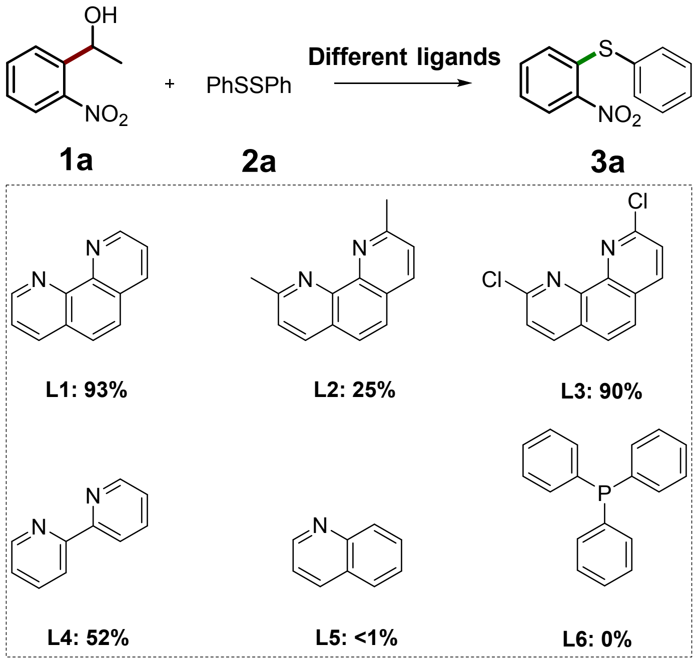

Figure S5. The Effect of different ligands on the C(aryl)−C bond thioetherification reaction. Reaction condition: 0.2 mmol substrate **1a**, 0.4 mmol diphenyldisulfane, 0.08 mmol $CuSO_4$, 0.04 mmol phen, 0.8 mmol K_2CO_3, 200 mg 4Å, 2 mL DMSO, 0.5 MPa O_2, 140 °C, 12 h.

*We also tested different organic ligands for the C(aryl)−C bond thioetherification reaction. Derivatives of phen ligands with electron-donating group Methyl **L2** significantly decreased the yield of thioether products. Bidentate ligand **L4** led to a moderate yield of 52%. Both monodentate N or P containing ligands (**L5** and **L6**) showed no activity for the reaction.*

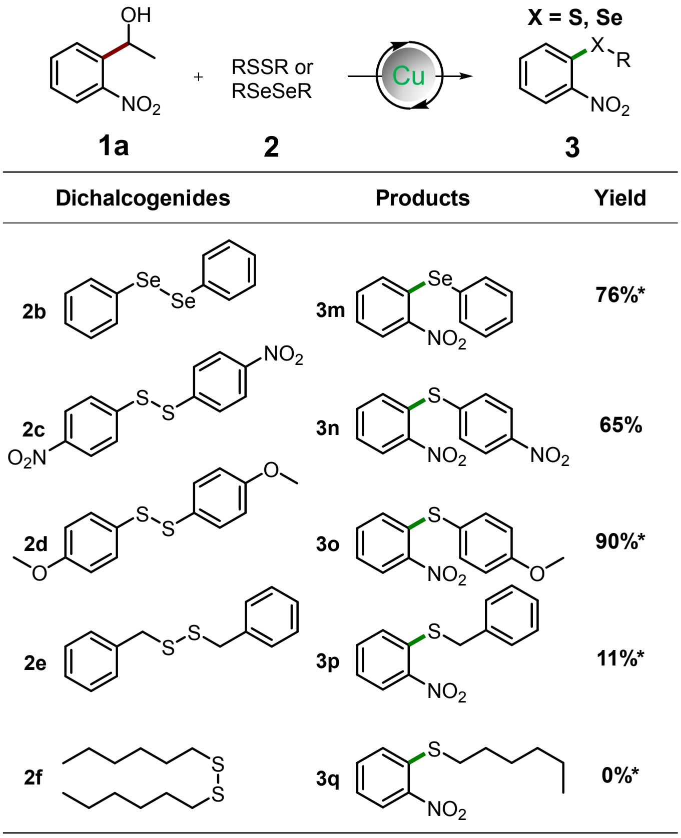

Figure S6. Substrate scope investigation using diphenyldisulfane derivatives and diphenyldiselane. Reaction condition: 0.2 mmol substrate **1a**, 0.4 mmol **diphenyldisulfane** or **diphenyldiselane**, 0.08 mmol $CuSO_4$, 0.04 mmol phen, 0.8 mmol K_2CO_3, 200 mg 4Å molecular sieve, 2 mL DMSO, 0.5 MPa O_2, 140 °C, 12 h, *24 h.

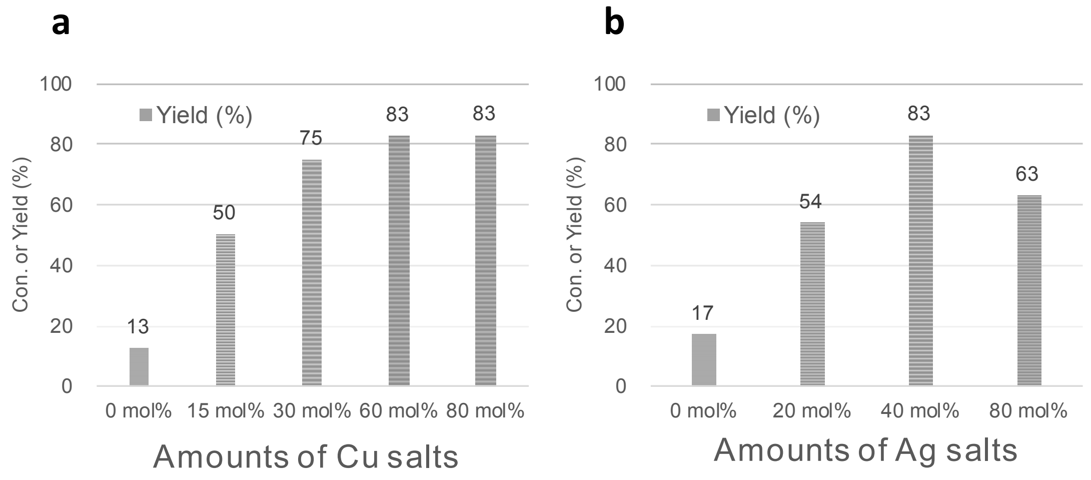

Figure S7. The effects of amounts of Cu salts and Ag salts on the C(aryl)−C bond hydrogenation reactions. Reaction condition **a**: 0.2 mmol substrate **1a**, **0-0.16 mmol CuCl**, 0.08 mmol $AgNO_3$, 0.8 mmol NaOH, 0.08 mmol phen, 2 mL DMSO, 0.5 MPa O_2, 140 °C, 8 h. Reaction condition **b**: 0.2 mmol substrate **1a**, 0.12 mmol CuCl, **0-0.16 mmol $AgNO_3$**, 0.8 mmol NaOH, 0.08 mmol phen, 2 mL DMSO, 0.5 MPa O_2, 140 °C, 8 h.

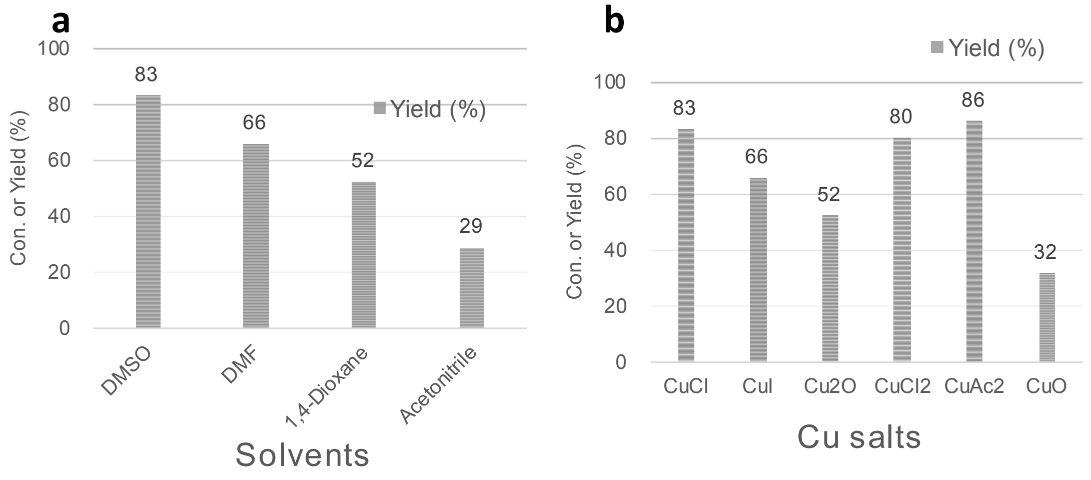

Figure S8. The effects of solvents and Cu salts on the C(aryl)−C bond hydrogenation reactions. Reaction condition **a**: 0.2 mmol substrate **1a**, 0.12 mmol CuCl, 0.08 mmol $AgNO_3$, 0.8 mmol NaOH, 0.08 mmol phen, **2 mL solvent**, 0.5 MPa O_2, 140 °C, 8 h. Reaction condition **b**: 0.2 mmol substrate **1a**, **0.12 mmol Cu salts**, 0.08 mmol $AgNO_3$, 0.8 mmol NaOH, 0.08 mmol phen, 2 mL DMSO, 0.5 MPa O_2, 140 °C, 8 h. $CuAc_2$: anhydrous copper acetate.

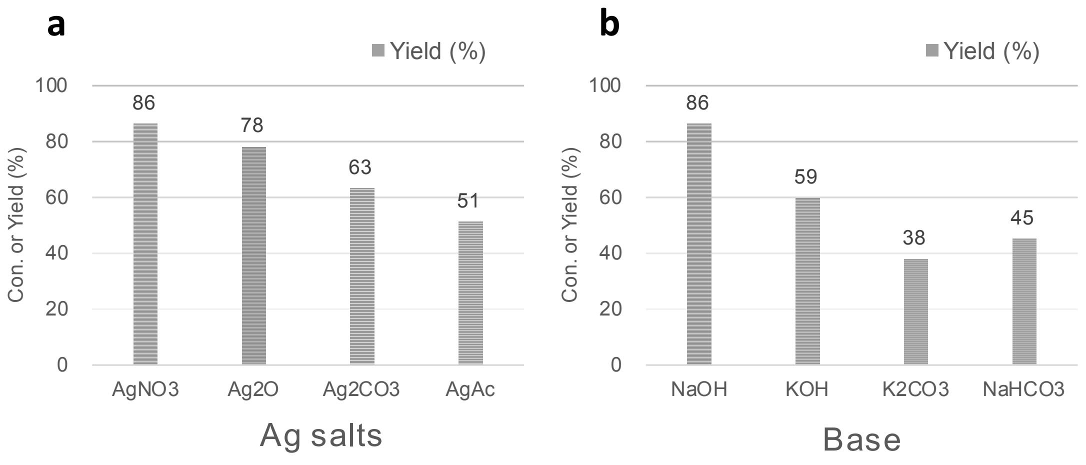

Figure S9. The effects of Ag salts and bases on the C(aryl)−C bond hydrogenation reactions. Reaction condition **a**: 0.2 mmol substrate **1a**, 0.12 mmol $CuAc_2$, **0.08 mmol Ag salts**, 0.8 mmol NaOH, 0.08 mmol phen, 2 mL DMSO, 0.5 MPa O_2, 140 °C, 8 h. Reaction condition **b**: 0.2 mmol substrate **1a**, 0.12 mmol $CuAc_2$, 0.08 mmol $AgNO_3$, **0.8 mmol base**, 0.08 mmol phen, 2 mL DMSO, 0.5 MPa O_2, 140 °C, 8 h.

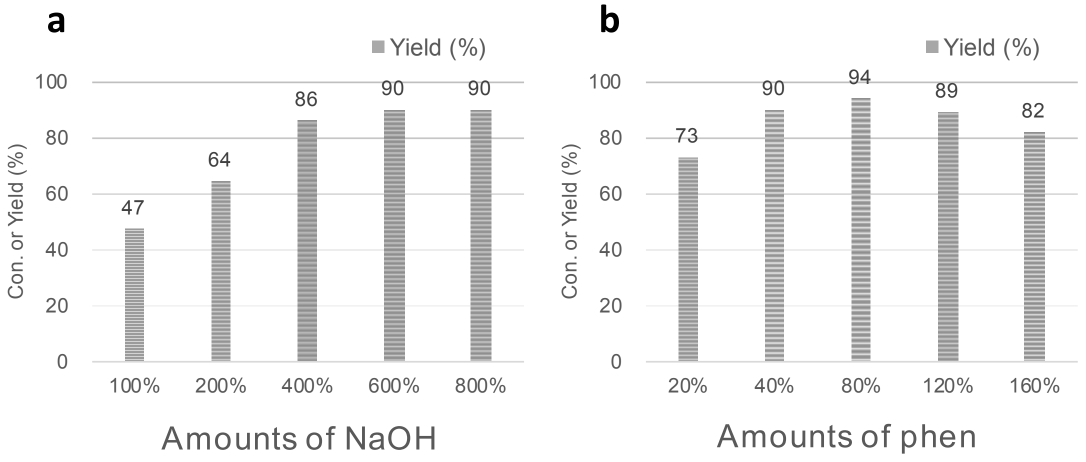

Figure S10. The effects of amounts of NaOH and phen on the C(aryl)−C bond hydrogenation reactions. Reaction condition **a**: 0.2 mmol substrate **1a**, 0.12 mmol $CuAc_2$, 0.08 mmol $AgNO_3$, **0.2-1.6 mmol NaOH**, 0.08 mmol phen, 2 mL DMSO, 0.5 MPa O_2, 140 °C, 8 h. Reaction condition **b**: 0.2 mmol substrate **1a**, 0.12 mmol $CuAc_2$, 0.08 mmol $AgNO_3$, 1.2 mmol NaOH, **0.04-0.32 mmol phen**, 2 mL DMSO, 0.5 MPa O_2, 140 °C, 8 h.

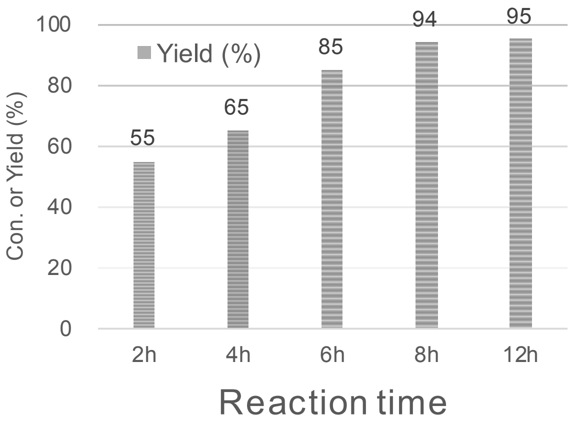

Figure S11. The effect of reaction time on the C(aryl)−C bond hydrogenation reactions. Reaction condition **a**: 0.2 mmol substrate **1a**, 0.12 mmol $CuAc_2$, 0.08 mmol $AgNO_3$, 1.2 mmol NaOH, 0.16 mmol phen, 2 mL DMSO, 0.5 MPa O_2, 140 °C, **2-12 h**.

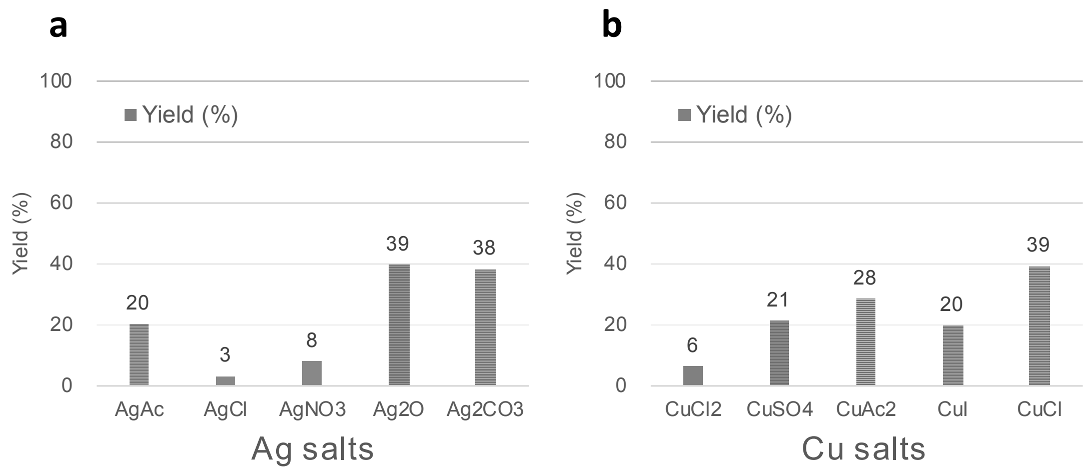

Figure S12. The effects of Ag slats and Cu salts on the C(aryl)−C bond carbonization reactions. Reaction condition **a**: 0.2 mmol **1a**, 0.5 mmol benzothiazole, 0.15 mmol CuCl, **0.25 mmol Ag salts**, 0.125 mmol phen, 0.2 mmol Cs_2CO_3, 200 mg 4Å, 2 mL DMF, 0.5 MPa O_2, 140 °C, 12 h. Reaction condition **b**: 0.2 mmol **1a**, 0.5 mmol benzothiazole, **0.15 mmol Cu salts**, 0.25 mmol Ag_2O, 0.125 mmol phen, 0.2 mmol Cs_2CO_3, 200 mg 4Å, 2 mL DMF, 0.5 MPa O_2, 140 °C, 12 h.

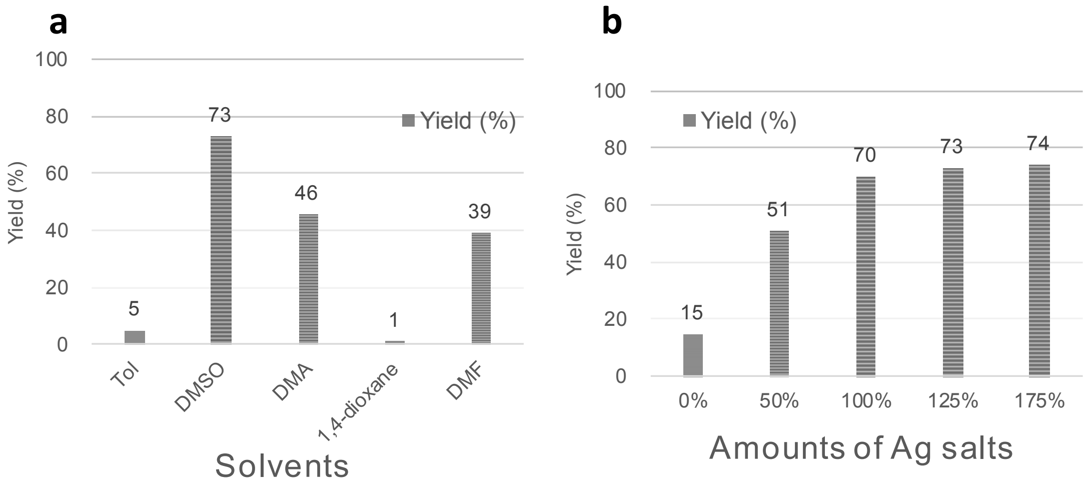

Figure S13. The effects of solvents and amounts of Ag_2O on the C(aryl)−C bond carbonization reactions. Reaction condition **a**: 0.2 mmol **1a**, 0.5 mmol benzothiazole, 0.15 mmol CuCl, 0.25 mmol Ag_2O, 0.125 mmol phen, 0.2 mmol Cs_2CO_3, 200 mg 4Å, **2 mL solvent**, 0.5 MPa O_2, 140 °C, 12 h. Reaction condition **b**: 0.2 mmol **1a**, 0.5 mmol benzothiazole, 0.15 mmol CuCl, **0-0.35 mmol Ag_2O**, 0.125 mmol phen, 0.2 mmol Cs_2CO_3, 200 mg 4Å, 2 mL DMSO, 0.5 MPa O_2, 140 °C, 12 h.

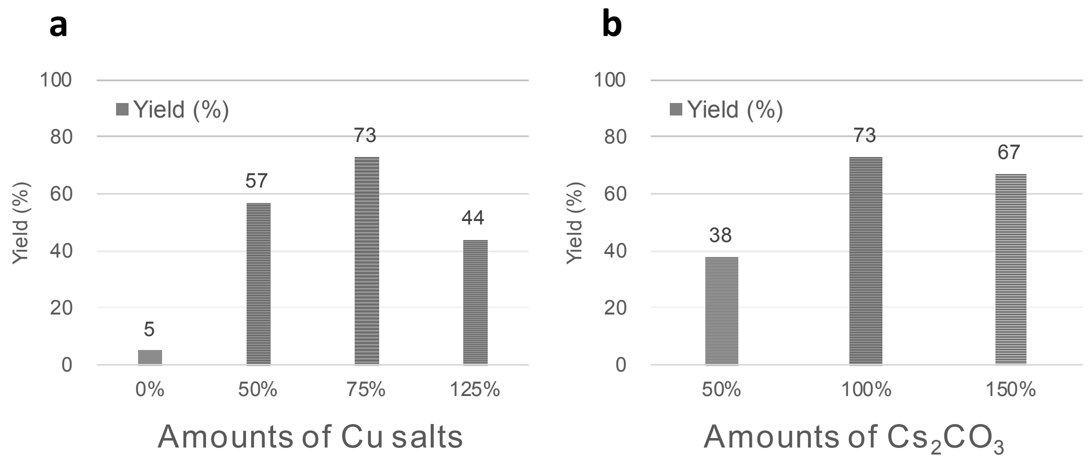

Figure S14. The effects of amounts of CuCl and Cs_2CO_3 on the C(aryl)−C bond carbonization reactions. Reaction condition **a**: 0.2 mmol **1a**, 0.5 mmol benzothiazole, **0-0.25 mmol CuCl**, 0.25 mmol Ag_2O, 0.125 mmol phen, 0.2 mmol Cs_2CO_3, 200 mg 4Å, 2 mL DMSO, 0.5 MPa O_2, 140 °C, 12 h. Reaction condition **b**: 0.2 mmol **1a**, 0.5 mmol benzothiazole, 0.15 mmol CuCl, 0.25 mmol Ag_2O, 0.125 mmol phen, **0.1-0.3 mmol Cs_2CO_3**, 200 mg 4Å, 2 mL DMSO, 0.5 MPa O_2, 140 °C, 12 h.

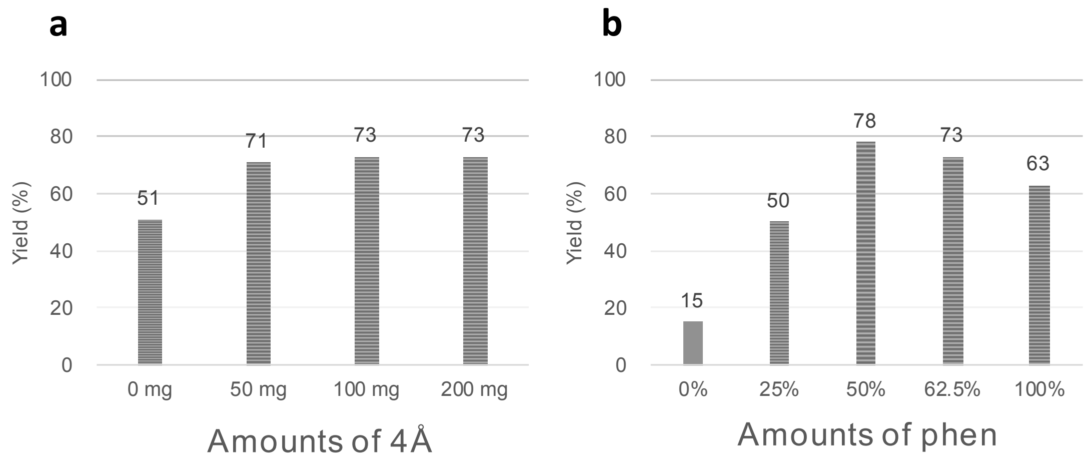

Figure S15. The effects of amounts of 4 Å and phen on the C(aryl)−C bond carbonization reactions. Reaction condition **a**: 0.2 mmol **1a**, 0.5 mmol benzothiazole, 0.15 mmol CuCl, 0.25 mmol Ag_2O, 0.125 mmol phen, 0.2 mmol Cs_2CO_3, **0-200 mg 4Å**, 2 mL DMSO, 0.5 MPa O_2, 140 °C, 12 h. Reaction condition **b**: 0.2 mmol **a**, 0.5 mmol benzothiazole, 0.15 mmol CuCl, 0.25 mmol Ag_2O, **0-0.2 mmol phen**, 0.2 mmol Cs_2CO_3, 200 mg 4Å, 2 mL DMSO, 0.5 MPa O_2, 140 °C, 12 h.

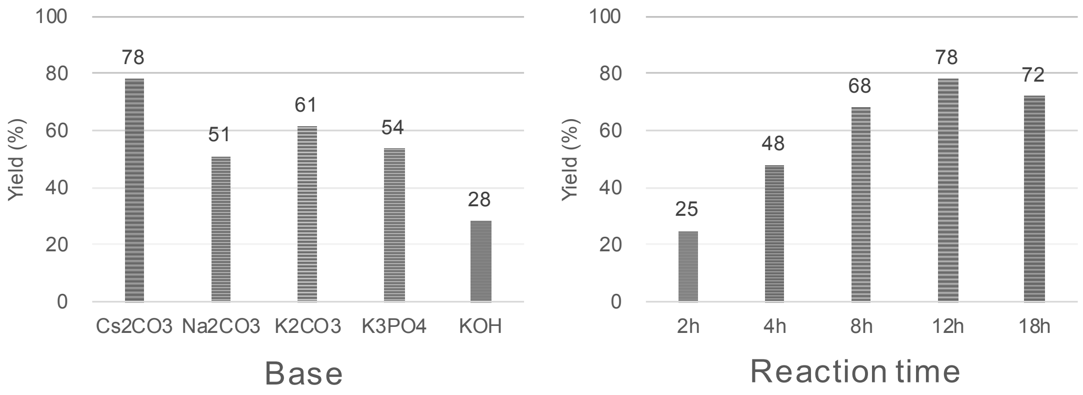

Figure S16. The effects of bases and reaction time on the C(aryl)−C bond carbonization reactions. Reaction condition **a**: 0.2 mmol **1a**, 0.5 mmol benzothiazole, 0.15 mmol CuCl, 0.25 mmol Ag_2O, 0.1 mmol phen, **0.2 mmol base**, 200 mg 4Å, 2 mL DMSO, 0.5 MPa O_2, 140 °C, 12 h. Reaction condition **b**: 0.2 mmol **a**, 0.5 mmol benzothiazole, 0.15 mmol CuCl, 0.25 mmol Ag_2O, 0.1 mmol phen, 0.2 mmol Cs_2CO_3, 200 mg 4Å, 2 mL DMSO, 0.5 MPa O_2, 140 °C, **2-12 h**.

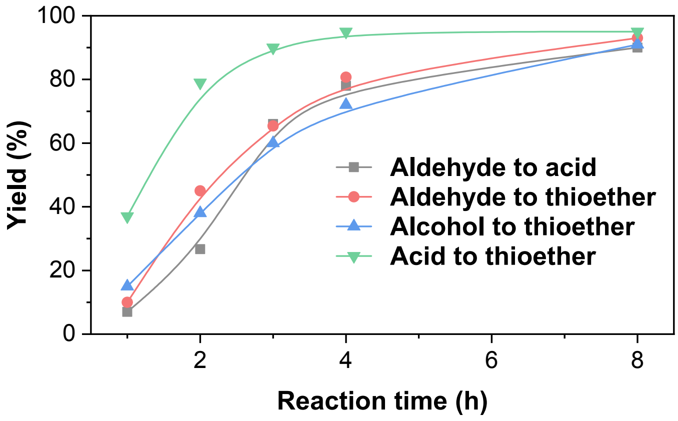

Figure S17. Kinetic study of the transformation of probable intermediates. We tested probable intermediates including aldehyde and acid derivatives of (1-(2-nitrophenyl)ethanol under the optimized conditions (as illustrated in Fig. 3b). The temporal evolution of corresponding products was monitored by GC. In comparison to the transformation of alcohol to thioether, aldehyde to thioether, and aldehyde to acid, the transformation rate of acid to thioether is obviously higher. Hence, the conversion of aldehyde to acid represents the rate-determining step in the reaction, rather than the decarboxylation step.

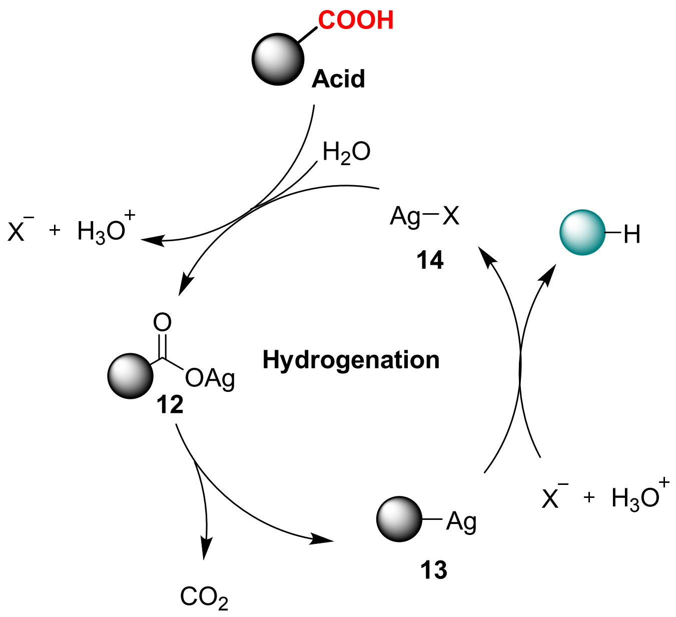

Figure S18. Plausible reaction pathway for the oxidative C(aryl)−C(OH) bonds hydrogenation reaction.

Starting from the acid intermediates, acid-base reaction between acid intermediates and Ag salts generates Ag carboxylate **12** followed by decarboxylation to aryl Ag species **13**. **13** is mediated with protodemetallation to afford the arene product. In comparison to Cu, Ag is a more efficient catalyst for decarboxylation. The presence of catalytic amount of Ag effectively promotes the decarboxylative hydrogenation of acid intermediates.[1-3]

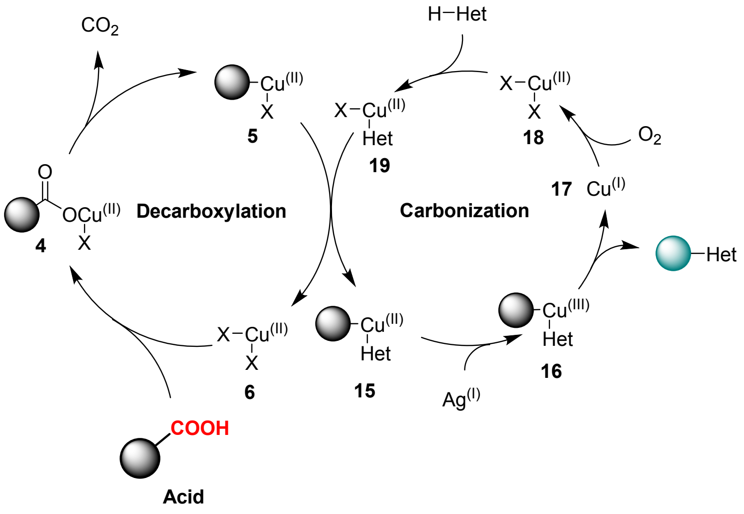

Figure S19. Plausible reaction pathway for the oxidative C(aryl)−C(OH) bonds carbonization reaction.

Starting from the acid intermediates, carbonization reaction is mediated with the same decarboxylation step for thioetherification reaction and generates intermediate **5**. **5** is further converted into a bifunctional aryl metal species **15** via transmetalation. The final products are generated by oxidation of **15** with Ag salts and reductive elimination of **16**. Stoichiometric amounts of Ag salt in carbonization reactions acts as a stronger oxidant than O_2 and oxidizes the $Cu^{(II)}$ **15** into $Cu^{(III)}$ **16**.[4-6]

Reference

1. J. Cornella, C. Sanchez, D. Banawa and I. Larrosa, *Chem. Commun.*, 2009, DOI: 10.1039/B916646G, 7176-7178.
2. P. Lu, C. Sanchez, J. Cornella and I. Larrosa, *Org. Lett.*, 2009, **11**, 5710-5713.
3. R. A. Crovak and J. M. Hoover, *J. Am. Chem. Soc.*, 2018, **140**, 2434-2437.
4. K. Xie, Z. Yang, X. Zhou, X. Li, S. Wang, Z. Tan, X. An and C.-C. Guo, *Org. Lett.*, 2010, **12**, 1564-1567.
5. L. Chen, L. Ju, K. A. Bustin and J. M. Hoover, *Chem. Commun.*, 2015, **51**, 15059-15062.
6. T. Patra, S. Nandi, S. K. Sahoo and D. Maiti, *Chem. Commun.*, 2016, **52**, 1432-1435.

NMR spectra

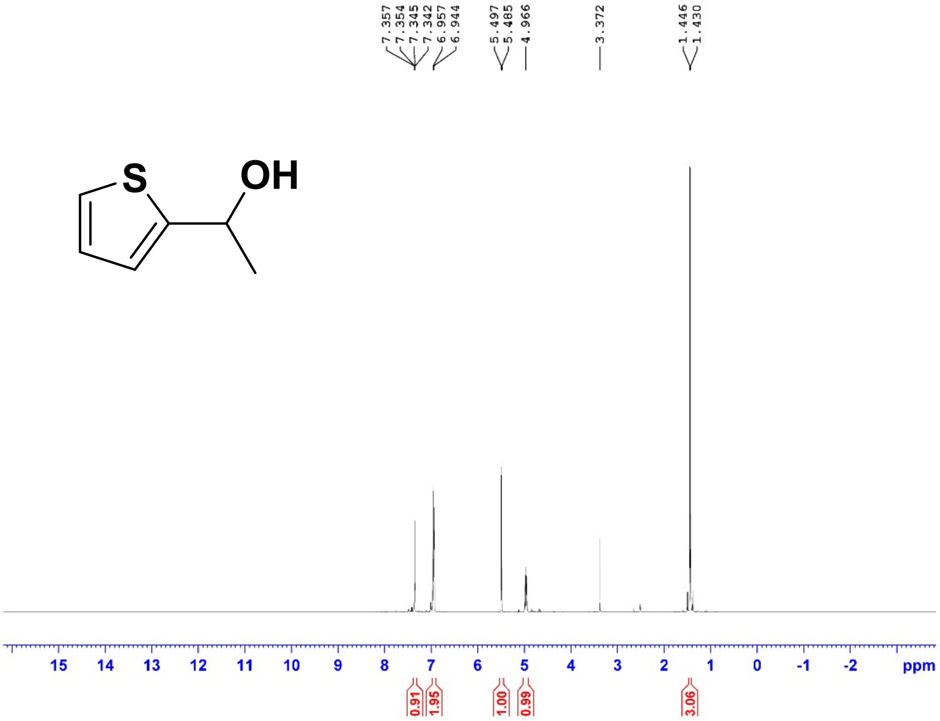

7.357
7.354
7.345
7.342
6.957
6.944
5.497
5.485
4.966
3.372
1.446
1.430
S
OH
15
14
13
12
11
10
9
8
7
6
5
4
3
2
1
0
-1
-2
ppm
0.91
1.95
1.00
0.99
3.06

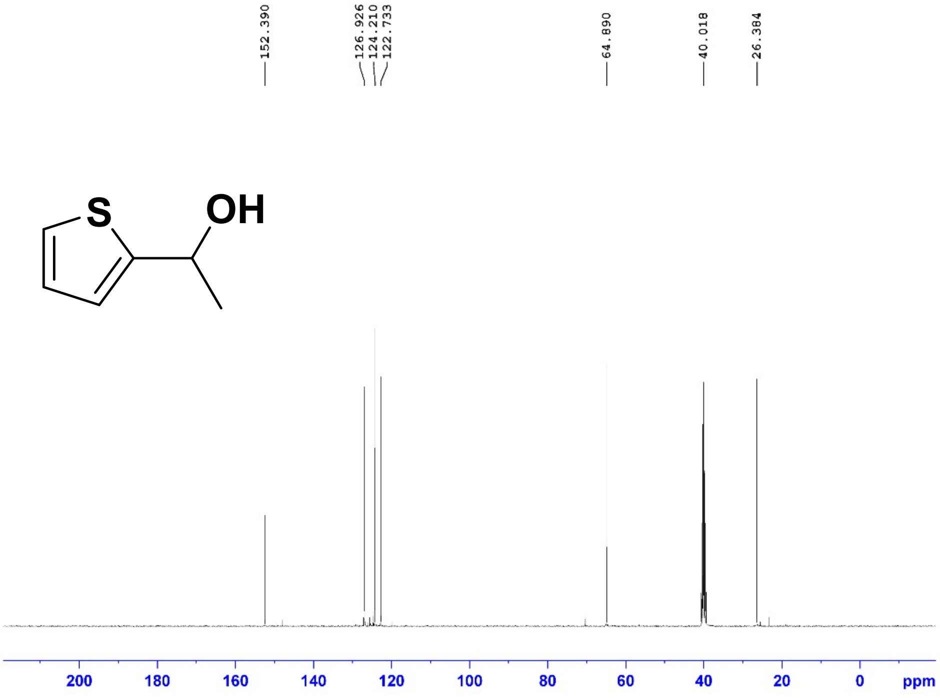

152.390
126.926
124.210
122.733
64.890
40.018
26.384
S
OH
200
180
160
140
120
100
80
60
40
20
0
ppm

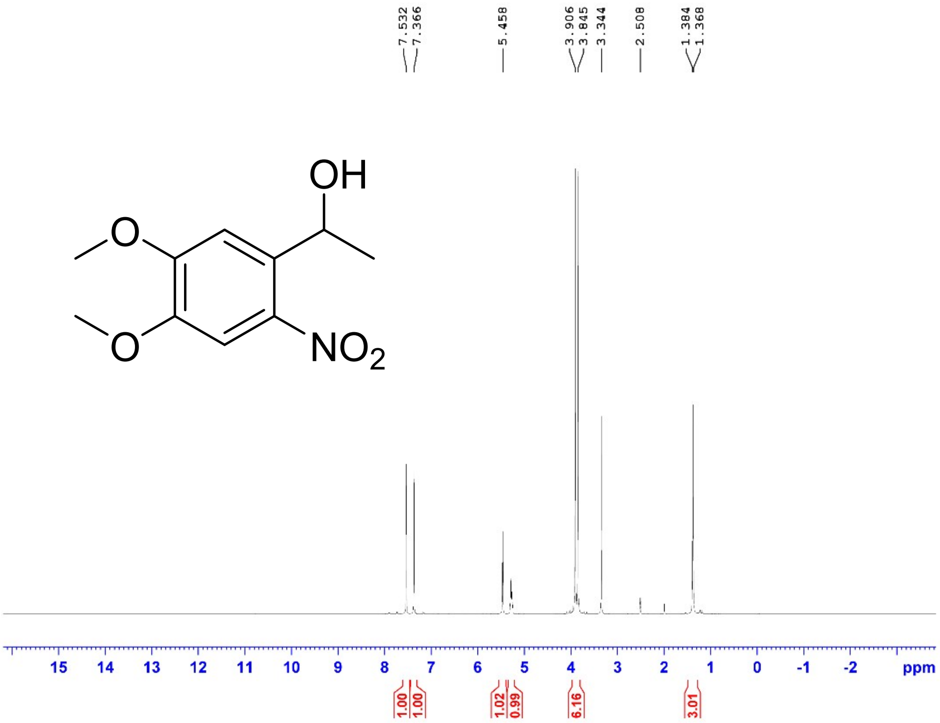
7.532
7.366
5.458
3.906
3.845
3.344
2.508
1.384
1.368
OH
O
O
NO2
15 14 13 12 11 10 9 8 7 6 5 4 3 2 1 0 -1 -2 ppm
1.00
1.00
1.02
0.99
6.16
3.01

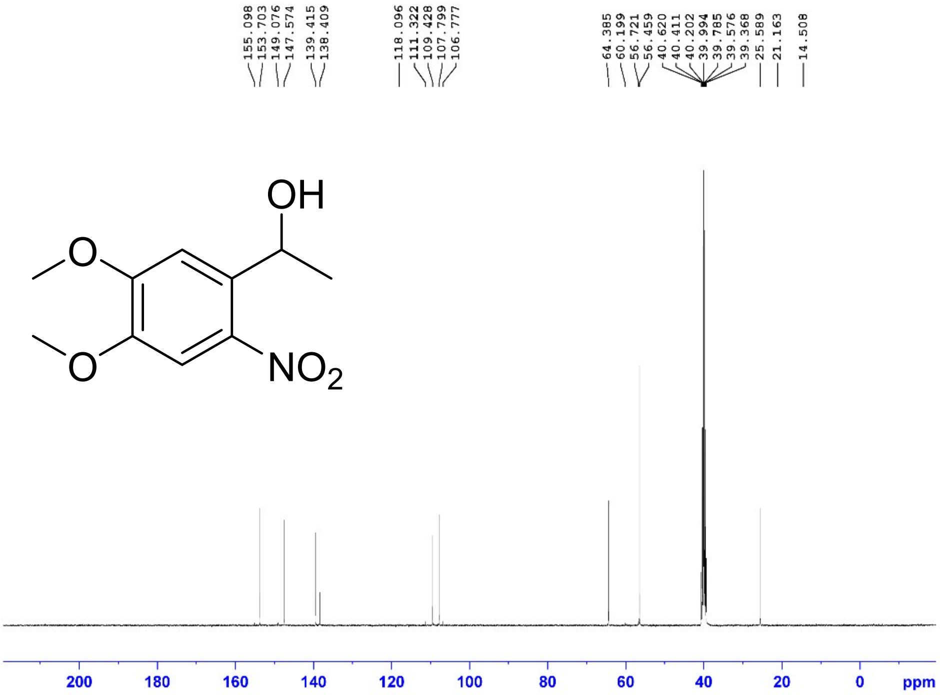
155.098
153.703
149.076
147.574
139.415
138.409
118.096
111.322
109.428
107.799
106.777
64.385
60.199
56.721
56.459
40.620
40.411
40.202
39.994
39.785
39.576
39.368
25.589
21.163
14.508
OH
O
O
NO2
200 180 160 140 120 100 80 60 40 20 0 ppm

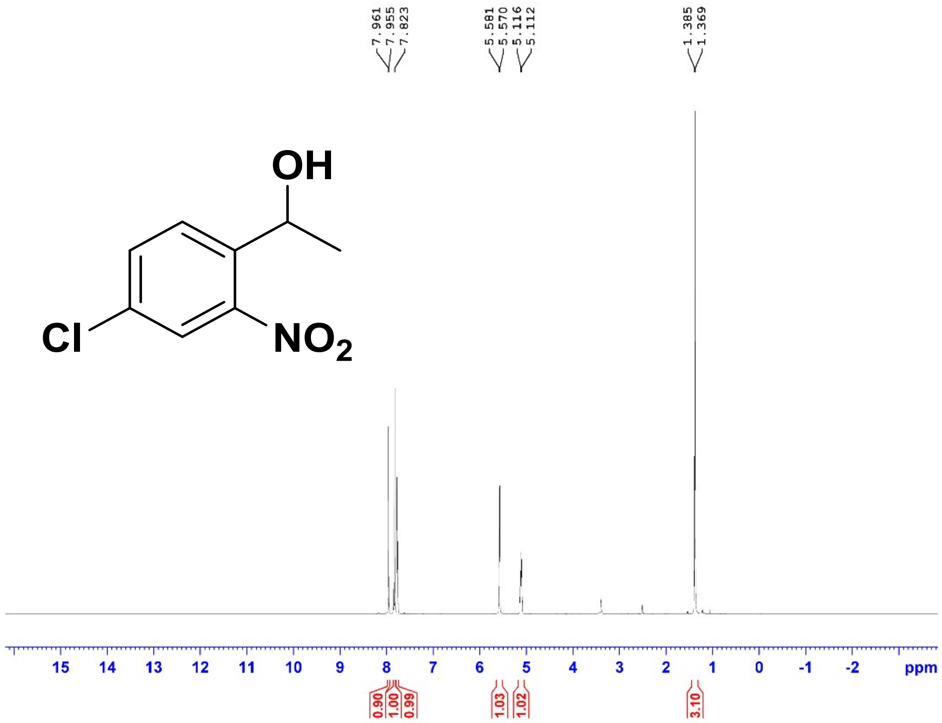

7.961
7.955
7.823
5.581
5.570
5.116
5.112
1.385
1.369
OH
Cl
NO2
15 14 13 12 11 10 9 8 7 6 5 4 3 2 1 0 -1 -2 ppm
0.90
1.00
0.99
1.03
1.02
3.10

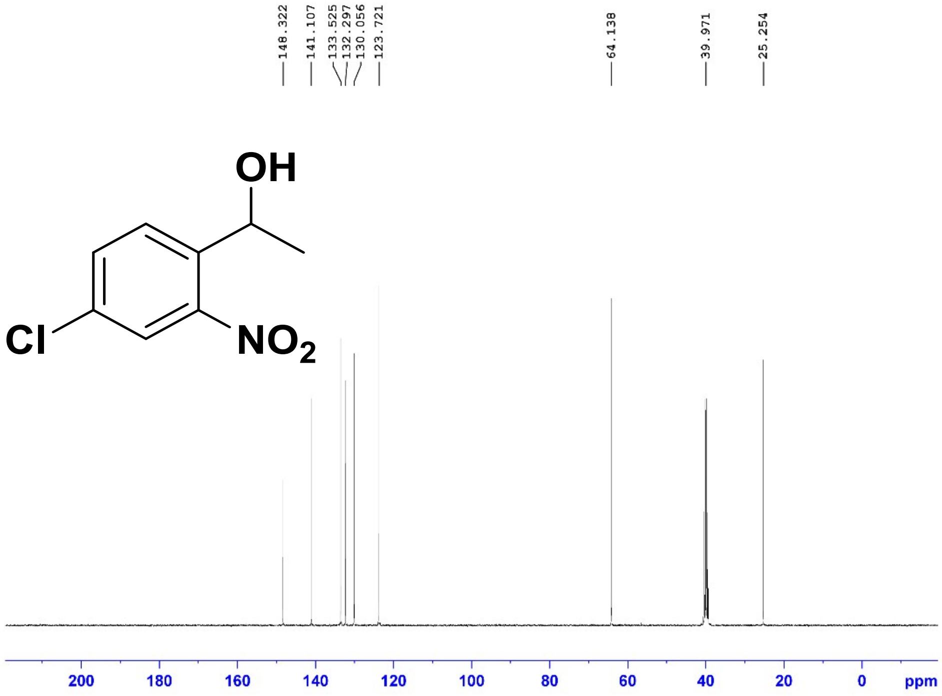

148.322
141.107
133.525
132.297
130.056
123.721
64.138
39.971
25.254
OH
Cl
NO2
200 180 160 140 120 100 80 60 40 20 0 ppm

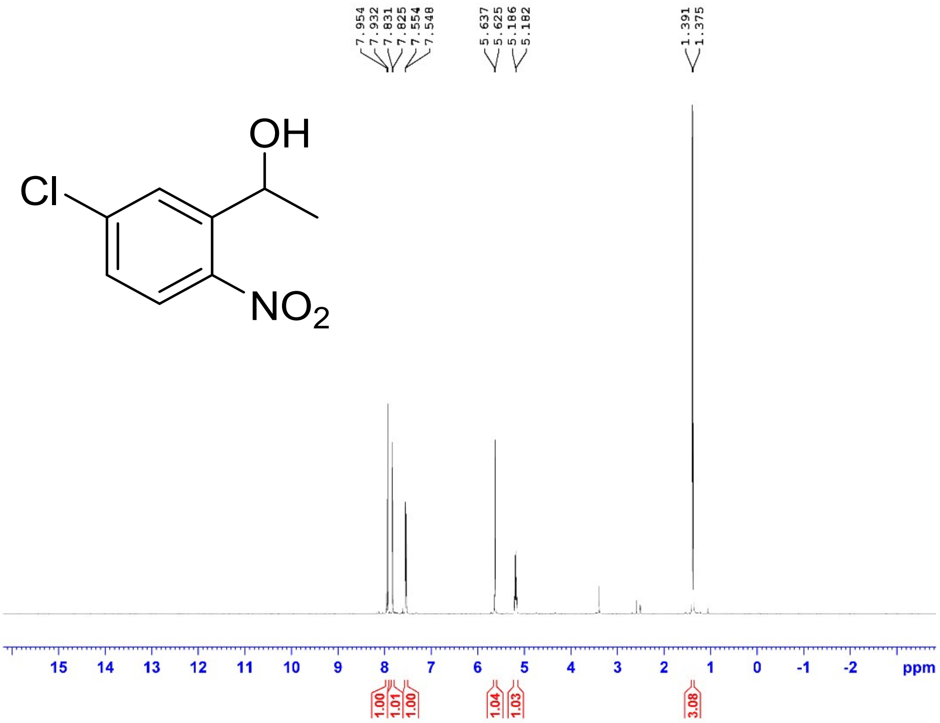
7.954
7.932
7.831
7.825
7.554
7.548
5.637
5.625
5.186
5.182
1.391
1.375
OH
Cl
NO2
15 14 13 12 11 10 9 8 7 6 5 4 3 2 1 0 -1 -2 ppm
1.00
1.01
1.00
1.04
1.03
3.08

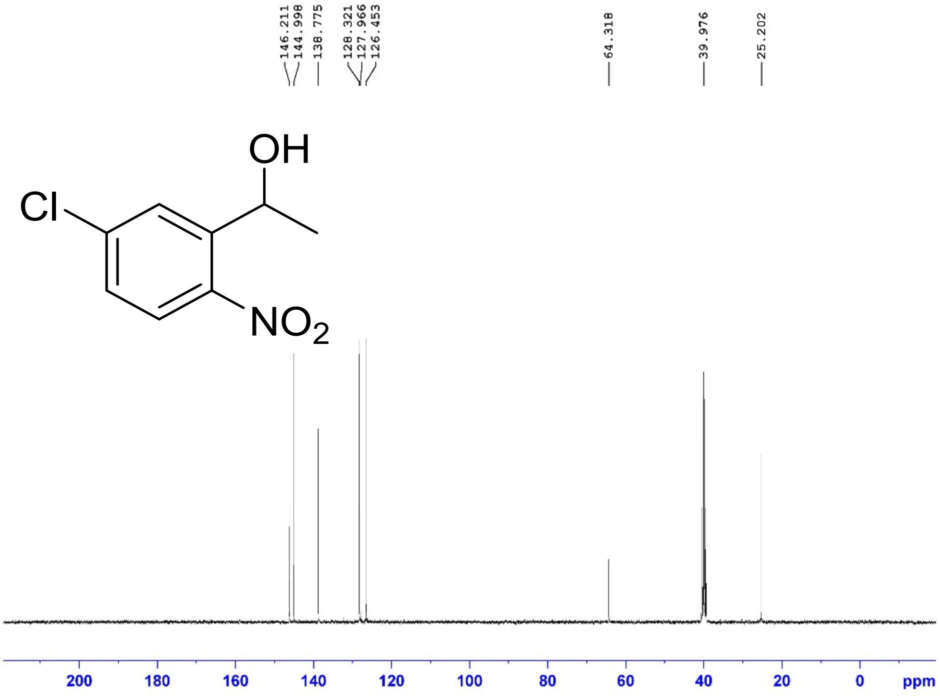
146.211
144.998
138.775
128.321
127.966
126.453
64.318
39.976
25.202
OH
Cl
NO2
200 180 160 140 120 100 80 60 40 20 0 ppm

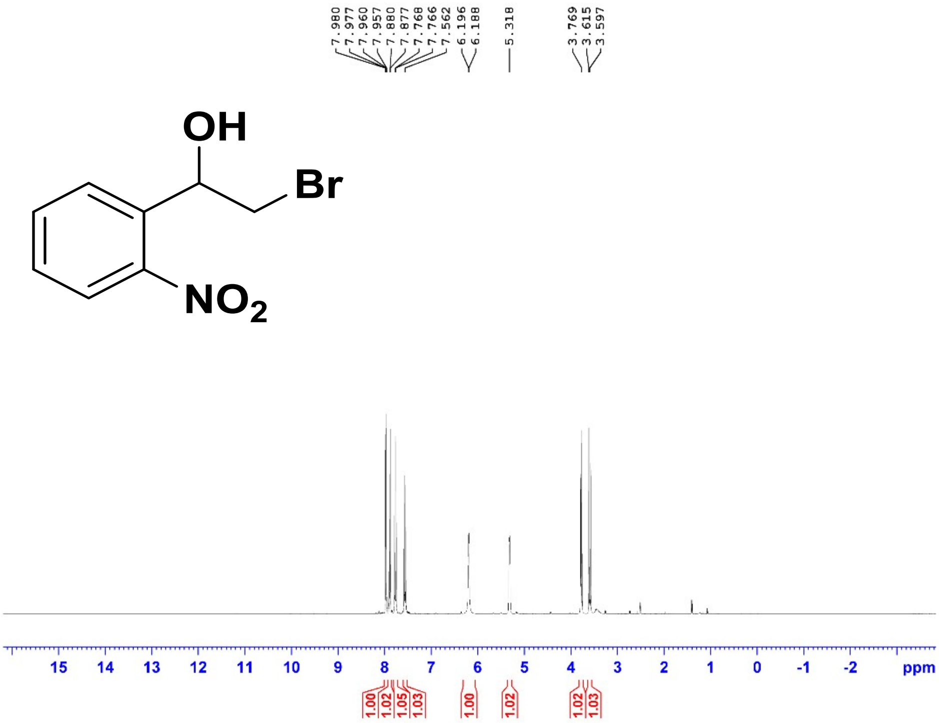
7.980
7.977
7.960
7.957
7.880
7.877
7.768
7.766
7.562
6.196
6.188
5.318
3.769
3.615
3.597
OH
Br
NO_2
15 14 13 12 11 10 9 8 7 6 5 4 3 2 1 0 -1 -2 ppm
1.00 1.02 1.05 1.03 1.00 1.02 1.02 1.03

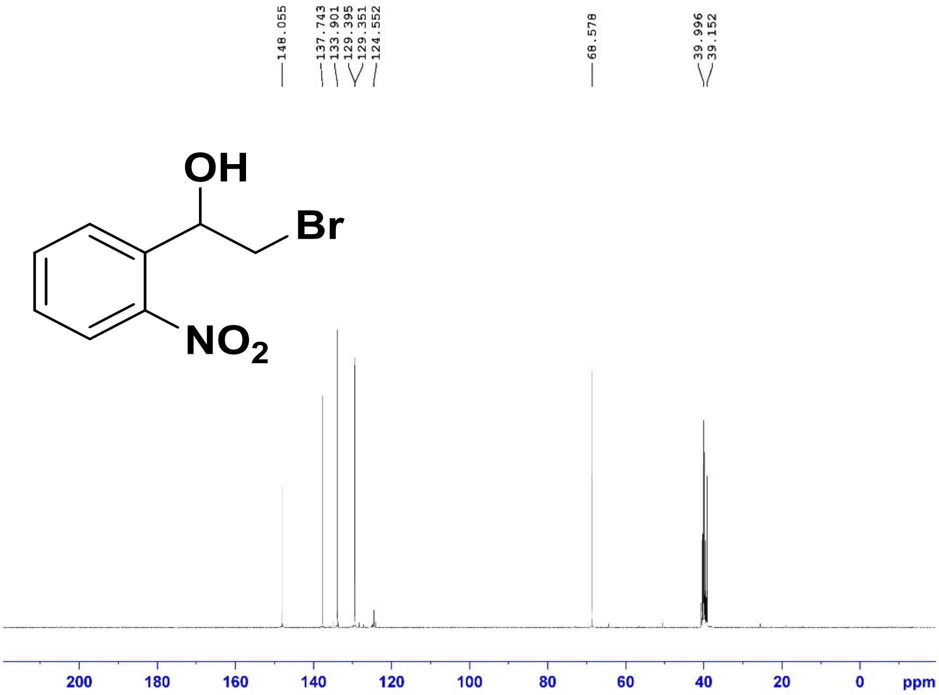
148.055
137.743
133.901
129.395
129.351
124.552
68.578
39.996
39.152
OH
Br
NO_2
200 180 160 140 120 100 80 60 40 20 0 ppm

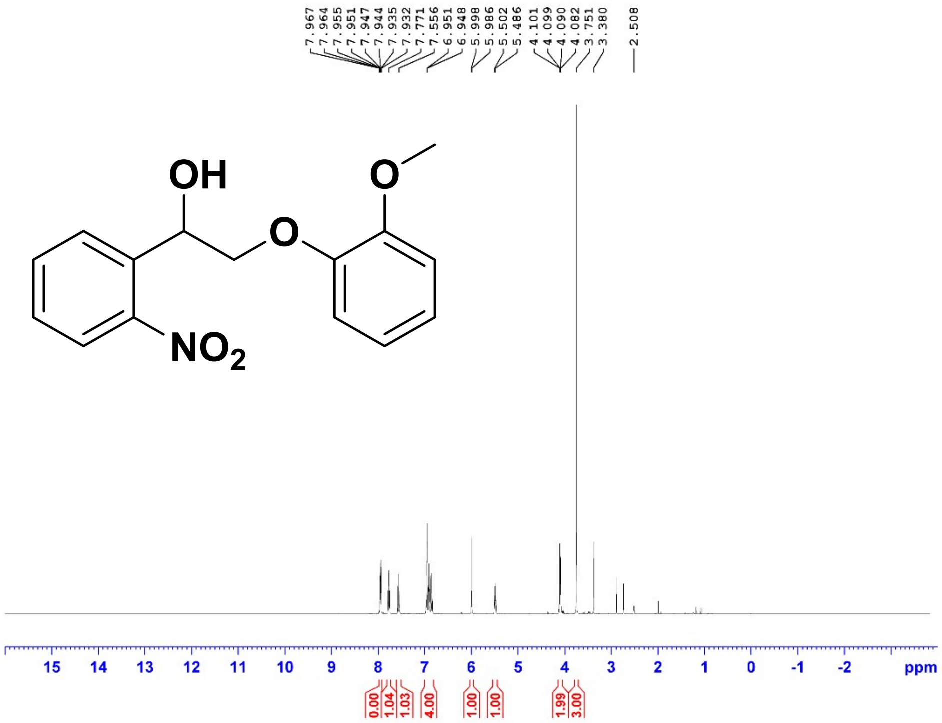

7.967 7.964 7.955 7.951 7.947 7.944 7.935 7.932 7.771 7.556 6.951 6.948 5.998 5.986 5.502 5.486 4.101 4.099 4.090 4.082 3.751 3.380 2.508
OH
O
O
NO2
15 14 13 12 11 10 9 8 7 6 5 4 3 2 1 0 -1 -2 ppm
0.00 1.04 1.03 4.00 1.00 1.00 1.99 3.00

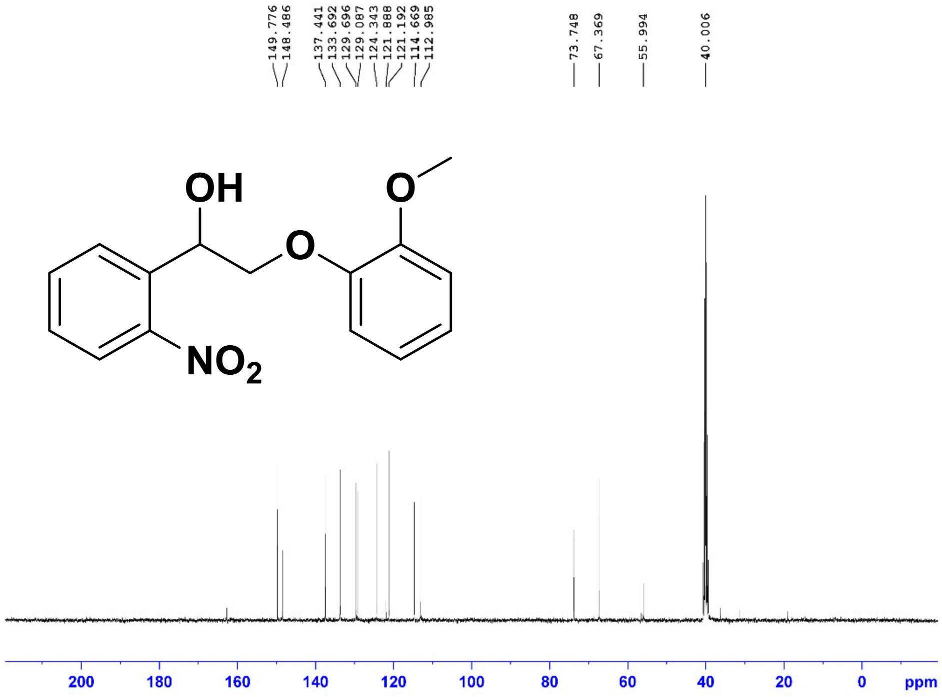

149.776 148.486 137.441 133.692 129.696 129.087 124.343 121.888 121.192 114.669 112.985 73.748 67.369 55.994 40.006
OH
O
O
NO2
200 180 160 140 120 100 80 60 40 20 0 ppm

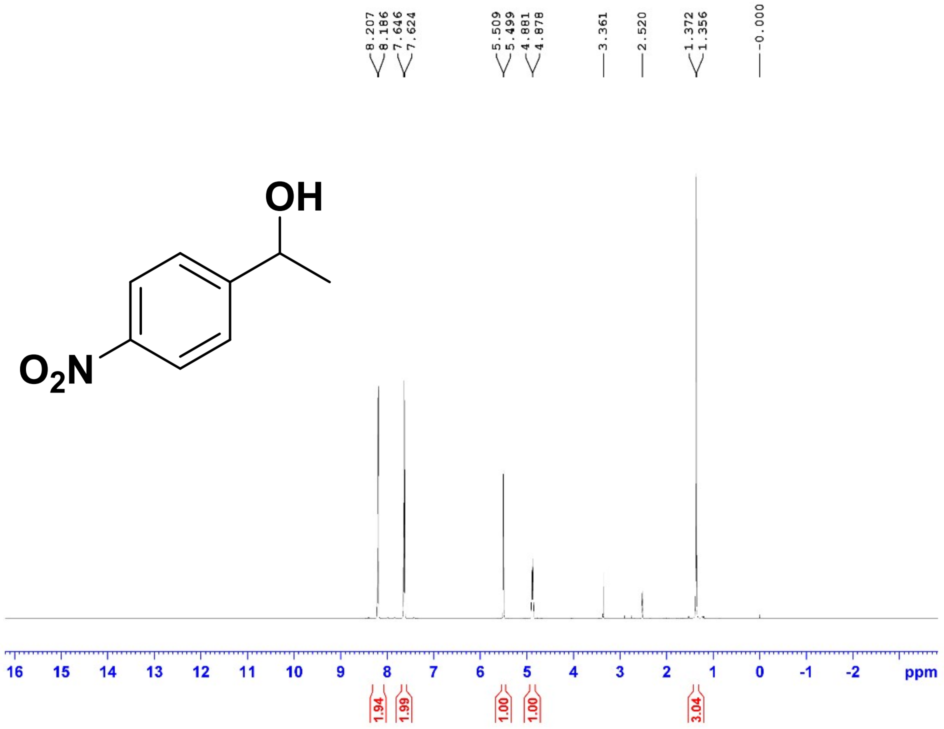

8.207
8.186
7.646
7.624
5.509
5.499
4.881
4.878
3.361
2.520
1.372
1.356
-0.000
OH
O2N
16 15 14 13 12 11 10 9 8 7 6 5 4 3 2 1 0 -1 -2 ppm
1.94
1.99
1.00
1.00
3.04

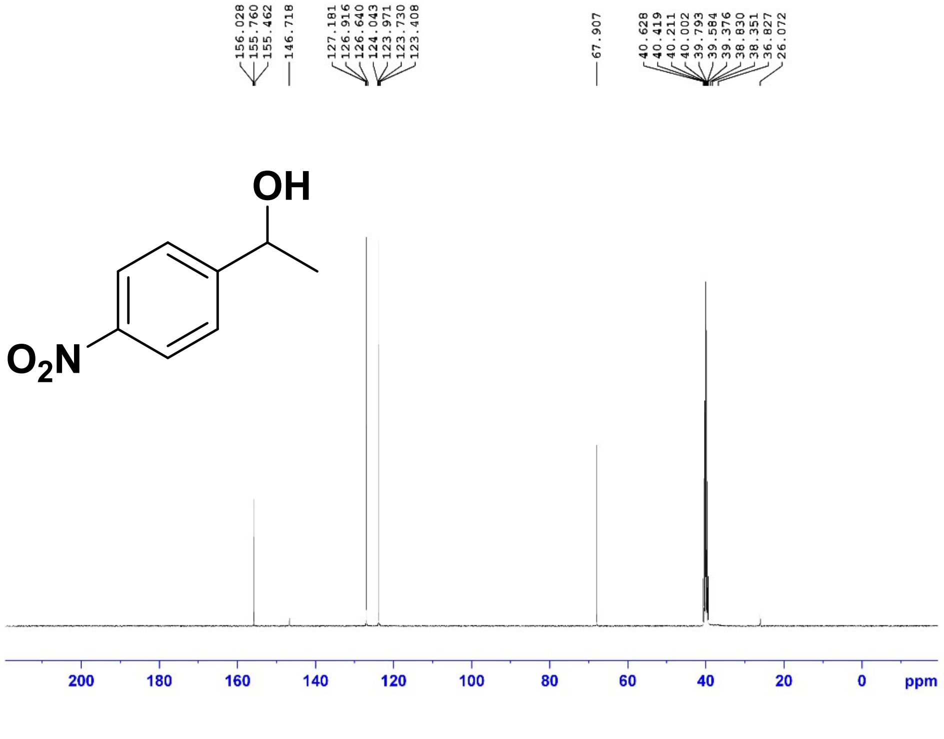

156.028
155.760
155.462
146.718
127.181
126.916
126.640
124.043
123.971
123.730
123.408
67.907
40.628
40.419
40.211
40.002
39.793
39.584
39.376
38.830
38.351
36.827
26.072
OH
O2N
200 180 160 140 120 100 80 60 40 20 0 ppm
